# Projecting the future spread of yellow fever vectors and implications for epidemic risk: a scoping review of climate change and human behaviour

**DOI:** 10.1136/bmjopen-2025-115595

**Published:** 2026-09-21

**Authors:** Andreza Parreiras-Gonçalves, Anna-Bella Failloux, Tomas Jelinek, Angelle Desiree LaBeaud, Edith Langevin, Eng Eong Ooi, Yaël Thollot, Oyewale Tomori, Nikos Vasilakis, Rachel Lowe, Felipe J Colón-González

**Affiliations:** 1 Laboratory of Immunology of Viral Diseases, Instituto René Rachou, Fundação Oswaldo Cruz (FIOCRUZ-Minas), Belo Horizonte, Brazil; 2 Institut Pasteur, Université Paris Cité, Arboviruses and Insect Vectors, Paris, France; 3 Berlin Centre for Travel and Tropical Medicine, Berlin, Germany; 4 Department of Pediatrics, Division of Infectious Disease, Stanford University School of Medicine, Stanford, California, USA; 5 Sanofi, Lyon, France; 6 Program in Emerging Infectious Diseases, Duke-NUS Medical School, , Singapore; 7 African Centre of Excellence for Genomics of Infectious Diseases, Redeemer’s University, Ede, Nigeria; 8 Department of Pathology and Center for Vector-Borne and Zoonotic Diseases, University of Texas Medical Branch, Galveston, Texas, USA; 9 Barcelona Supercomputing Center (BSC), Barcelona, Spain; 10 Catalan Institution for Research & Advanced Studies (ICREA), Barcelona, Spain; 11 Centre on Climate Change & Planetary Health and Centre for the Mathematical Modelling of Infectious Diseases, London School of Hygiene & Tropical Medicine, London, UK; 12 Data for Science and Health, Wellcome Trust, London, UK

**Keywords:** VIROLOGY, Climate Change, Mosquito Vectors, Tropical medicine

## Abstract

**Abstract:**

**Background:**

Yellow fever is a vector-borne disease with high epidemic potential. Although currently concentrated in Africa and Latin America, both climate change and human behaviour are likely to affect its future spread and epidemic risk. However, how these factors might interact to alter yellow fever distribution remains unclear.

**Objectives:**

This scoping review aimed to map the available literature at the intersection of these topics and compile the trends that may influence the future spread of yellow fever.

**Eligibility criteria:**

Studies were included if they reported original data on future trends in yellow fever epidemiology or vector evolution in the context of climate change, climate variability and human behaviour. Review articles, reports without original data, reports not addressing future trends in yellow fever epidemiology or vector evolution in the context of climatic factors or human behaviour were excluded.

**Sources of evidence:**

Systematic searches of PubMed, Embase and Cochrane CENTRAL were conducted from database inception up to September 2024, alongside manual searches of congress abstract books conducted in September 2024.

**Charting methods:**

The project followed Joanna Briggs Institute methodology for scoping reviews, using Preferred Reporting Items for Systematic Reviews and Meta-Analyses extension for Scoping Reviews guidelines. Searches focused on publications containing data relating to predicting, modelling or forecasting trends over time in yellow fever disease, virus and vector dynamics in relation to climatic factors and/or human behaviour. Search results underwent two-step screening against inclusion and exclusion criteria. Data were manually extracted using a bespoke Excel form, with results classified according to expert opinion. Trends in the dataset were identified using generative artificial intelligence as a drafting aid and all final categorisation and interpretation was determined through expert manual review.

**Results:**

In all, 1243 studies were screened and 42 met inclusion criteria. The oldest study was published in 2009, and 67% were published between 2019 and 2024. Most (81%) were modelling studies. Most included studies projected a climate-driven expansion in the range and abundance of major yellow fever vectors, particularly *Aedes aegypti*, with temperature and precipitation the most frequently examined covariates. Human factors such as urbanisation and population growth were also commonly modelled as amplifiers of exposure risk. Evidence directly linking these projections to future yellow fever transmission was less frequent and more heterogenous. Important knowledge gaps at this topic intersection included limited research on non-*Aedes* vectors, insufficient information on future yellow fever trends in Africa and limited understanding of how sylvatic cycles might be affected by climate change and human behaviour.

**Conclusions:**

The available literature suggests that changing climatic conditions and human activities such as urbanisation and land use will likely contribute to future increases in yellow fever vector abundance and geographical range. While most relevant analyses suggest that increases in human exposure and transmission risk are likely to follow, evidence related to these factors was more limited.

**STRENGTHS AND LIMITATIONS OF THIS STUDY**

- The study followed established Joanna Briggs Institute methodology and Preferred Reporting Items for Systematic Reviews and Meta-Analyses extension for Scoping Reviews reporting guidelines, with a registered protocol (INPLASY).
- The results of the systematic literature search and data extraction used a hybrid approach combining the use of AI-assisted summarisation followed by full expert review, which improved efficiency while adding an additional interpretive step.
- As is typical for scoping reviews, no formal critical appraisal of included studies was undertaken, limiting inferences about the comparative strength of evidence.
- Focusing the search on studies assessing future trends may have resulted in the omission of studies on aspects of human behaviour, which are difficult to model.

## Introduction

 Yellow fever (YF) is among the most serious of mosquito-borne diseases due to its ability to spark major epidemics. Although currently concentrated in Africa and Latin America, YF affected North America and Europe until the early 20th century.^1^ The yellow fever virus (YFV, *Orthoflavivirus flavi*) is an orthoflavivirus (*Flaviviridae* family, genus *Orthoflavivirus*) and is transmitted to humans by the bites of infected *Aedes, Haemagogus* or *Sabethes* spp. mosquitoes.^2^ YFV has a sylvatic cycle in which it circulates in non-human primates (NHPs) and arboreal *Haemagogus* and *Sabethes* spp, but it can also circulate in intermediate (rural) and urban cycles. The main urban vector is *Aedes aegypti*. Transmission of YFV by *Ae. aegypti* can occur either via spillover from NHPs to humans or from human to human, the latter being responsible for urban YF epidemics. For the past few decades, the urban cycle has been reported only in Africa, while all cases in South America have been related to the sylvatic cycle and spillover events into rural and periurban settings. The main rural and periurban vectors in the Latin American region are *Haemagogus* and *Sabethes* species, which mediate the sylvatic cycle, as highlighted earlier.^3 4^

In 2023, 30 countries in Africa and 13 countries in the Americas were listed by the WHO as being at risk of YF transmission.^5^ It is estimated that YF caused approximately 109 000 severe infections and 51 000 deaths in Africa and South America in 2018.^6^ Underreporting remains a concern, and the true number of cases may be 10 to 250 times higher than currently recorded.^2^ In 2024, a total of 447 human cases of YF were reported to WHO from 16 countries in Africa.^7^ In the Americas, 61 human YF cases were recorded in 2024, of which 30 were fatal.^8^ In 2025, there were 346 recorded cases in seven countries in the same region, with 148 fatal cases.^8^

Although effective vaccines for YF exist, current manufacturing capacities are unlikely to meet demand if an outbreak of similar magnitude to those in the recent past were to occur now. Many locations vulnerable to YF introduction are also endemic for other arboviruses such as dengue virus (*Orthoflavivirus denguei*), Zika virus (*Orthoflavivirus zikaense*), Japanese encephalitis virus (*Orthoflavivirus japonicum*) and chikungunya virus (*Alphavirus chikungunya*). Misdiagnosis of YF as dengue fever due to shared early symptoms and serological cross-reactivity may delay effective public health responses and exacerbate YF outbreaks.^9^

Various factors can influence the distribution and spread of YF, including insufficient vaccination coverage, virus and vector adaptation, and failures in vector control.^10–12^ Variation in climate conditions is one such factor affecting YF spread. This includes both long-term alteration of climate conditions (‘climate change’, primarily caused by anthropogenic greenhouse gas emissions from human activity) and more short-term fluctuations over a period of seasons, years or decades (‘climate variability’, caused both by natural processes and contributions from human activity). Extreme climatic events, exacerbated by climate change, are expected to lead to more frequent severe heatwaves, droughts, floods and storms. In addition, natural variations such as the El Niño-Southern Oscillation cause irregular disruption to normal weather patterns.

Climatic factors may also influence other critical aspects of YF, such as the distribution and abundance of animal reservoirs, the survival and life cycle of vectors, and the severity of outbreaks.^13 14^ Temperature changes may alter vector distribution,^12 13^ with *Ae. aegypti* likely to expand geographically as global temperatures rise.^15–17^ When vector population density increases, this increases the likelihood of YFV transmission and thereby YF.^18^

Human activities can directly facilitate the range expansion of YF vectors, for example, by providing potential breeding sites through domestic water storage. Human population centres close to forested areas increase the frequency of contact between humans, NHPs and vectors. International travel facilitates the movement of infected individuals and hence the importation of YF into non-endemic areas, illustrated by the cluster of YF cases in China caused by eleven Chinese workers returning from Angola during the 2016 outbreak.^19^

Human activities can also help to reduce the incidence of the disease, for example, through vaccination policies and vector control. All YF epidemics in the Americas since the second half of the 20th century have resulted from sylvatic spillover, partially due to the fact that the main urban vector, *Ae. aegypti*, was targeted by nationwide eradication programmes in the 1930s, 1940s and 1950s.^20^ While *Ae. aegypti* populations were reestablished from the 1960s onwards, they have been notably uninvolved in outbreaks in South America during this period.^20 21^ Furthermore, sustained urban transmission is unlikely in the presence of robust YFV vaccination coverage.^22 23^ Nevertheless, health systems and vaccination policies can vary greatly from one region to another, and where susceptible individuals remain unvaccinated, spillover events can still lead to outbreaks, such as the outbreak in Brazil in 2016–2018. The risk is concentrated among unvaccinated individuals, who account for the majority of YF outbreak cases. Human behaviour in the context of YF also encompasses broader social and national dimensions, including vaccination policy, healthcare infrastructure and community engagement in vector control, which interact with environmental and climatic drivers to shape YF risk across regions. Here, human behaviour refers to human-driven demographic, mobility, land-use, vaccination and vector-control factors that may interact with environmental change and alter vector range, human exposure or transmission dynamics.

Exactly how climatic conditions and human behaviour might interact to influence the distribution of YF vectors and transmission of YFV remains unclear, and prediction of YF spread is inherently complex. This uncertainty is illustrated by several unresolved patterns in the current distribution of YF and its vectors. For example, *Ae. aegypti* has been slow to re-establish itself in Europe, particularly southern Europe (where it was previously established until the middle of the 20th century), despite apparently favourable environmental conditions.^24–26^ Similarly, YF has not yet become established in Asia or the Pacific region, despite widespread infestation with *Ae. aegypti* and suitable climatic conditions; competition with other viruses has been suggested as a possible explanation.^27–29^ These observations highlight the difficulty of predicting YF spread from vector presence or climate suitability alone and suggest that future work must better incorporate climatic, ecological and human behavioural drivers within an integrated framework.

The aim of this scoping review was to explore the future spread and epidemic risk of YF in the context of climate change and/or human behaviour by screening the available literature to identify consensus trends and knowledge gaps.

## Methods

### Study design

The scoping review was conducted in accordance with the Joanna Briggs Institute (JBI) methodology for scoping reviews.^30^ The study was guided by the Preferred Reporting Items for Systematic Reviews and Meta-Analyses Extension for Scoping Reviews (PRISMA-ScR).^31^ The patients, exposure and outcomes (PEO) conceptual framework was used to frame the inclusion and exclusion criteria for the review and consisted of the population of interest (studies reporting YF epidemiology/vector evolution) and the factors (climate change, climate variability and human behaviour) that affect the outcomes of interest (future epidemiological and vector evolution trends). All studies reporting original data relating to future trends of YF epidemiology/vector evolution in the context of climate change, climate variability and human behaviour were included; review articles and reports lacking original data or which did not contain data about future trends in YF epidemiology or vector evolution in the context of climatic factors or human behaviour were excluded. Publications only relating to *Aedes albopictus* were excluded as it was not regarded by entomologists as an official vector of YF at the time of the analysis (table 1). The exact search terms used to shape the operational definitions of ‘climate change’ and ‘human behaviour’ are in the online supplemental material protocol S1. The scoping review was based on an existing protocol registered at the International Platform of Registered Systematic Review and Meta-analysis Protocols (INPLASY) under protocol number INPLASY2024110098^32^; reporting was in accordance with the PRISMA-ScR (online supplemental material checklist S1).

**Table 1:** Publication inclusion and exclusion criteria

Criteria categories	Inclusion criteria	Exclusion criteria
Participants / populations	All studies reporting YFV, disease or vector evolution will be considered. Human or non-human studies.	
Outcomes	Publications containing data relating to predicting / modelling / forecasting trends over time of: YFV and YF disease (eg, burden of disease, number of human / non-human cases, disease severity and mortality and geographical distribution) YF vector (*Aedes*, *Haemagogus* or *Sabethes* mosquitoes) evolution, changes in mosquito development / distribution, changes in YF transmission in relation to climatic factors and/or in relation to human behaviour.	Publications containing no data related to **future trends** of YF epidemiology or vector evolution in relation to climatic factors or human behaviour. Publications on vaccine efficacy. Publications only relating to *Ae. albopictus*.
Publication types	Journal articles. Congress abstracts.	Review articles with no original data. Preprints.
Language	No restriction on language.	
Publication timeframe	All evidence available up to the time of the final literature searches.	
Publication status	Published and unpublished literature including congress abstracts.	

YF, yellow fever; YFV, yellow fever virus.

### Patient and public involvement

No patients or members of the public were involved in the study design, conduct, reporting or dissemination plans of this research.

### Search strategy

The search strategy was designed in conjunction with an information professional to identify publications containing data relating to predicting / modelling / forecasting trends over time of YFV and YF disease (eg, burden of disease, number of human/non-human cases, disease severity and mortality and geographical distribution), YF vector (*Aedes*, *Haemagogus* or *Sabethes* mosquitoes) evolution, changes in mosquito development / distribution and changes in YF transmission in relation to climatic factors and/or in relation to human behaviour. Relevant terms (including synonyms, abbreviations, variant spellings, etc.) and index terms such as Medical Subject Headings (MeSH) and Emtree were specified, and Boolean operators were used to create search queries (online supplemental material protocol S1). Searches were conducted from database inception up to 11 September 2024 in the following databases: (1) MEDLINE via PubMed, (2) Embase via Embase.com, (3) Cochrane CENTRAL via the Cochrane Library. Manual searches were conducted on 13 September 2024 of congress abstract books (last 2 years of *Conference of the International Society of Travel Medicine* (*CISTM*), *American Society of Tropical Medicine & Hygiene Congress* (*ASTMH*), *Pan-American Dengue Research Network* (*PanDengue Net*) *Meeting*) to identify additional studies. *The Journal of Climate Change and Health* was also searched manually for additional relevant articles. These conference proceedings and journal sources were selected because they were judged most likely to capture recent projection-focused work on YF and mosquito ecology that might not yet be fully indexed in bibliographic databases. Finally, the reference lists of key studies were checked to identify additional relevant studies for inclusion.

### Screening

All search results were uploaded to EndNote (Clarivate Analysis, PA, USA), and duplicates were removed. The final set of results was uploaded into the review management tool Covidence (Veritas Health Innovation Ltd. VIC, Australia) for two-step screening by two independent reviewers for assessment against the inclusion criteria. In the first step, studies were screened based on their title and abstract; if inclusion criteria were met, then full-text versions were obtained and linked to the relevant citation within Covidence. In the second step, the full-text versions were screened against the inclusion criteria. Reasons for exclusion were recorded; studies that focused only on non-YF vectors were excluded. Any disagreements between the reviewers at each screening round were resolved through discussion or with a senior member of the review team.

### Data extraction

A bespoke data extraction form was developed in Excel by the review team (online supplemental material protocol S2). Specific details about the focus (including vector, methodology, analysis and covariates), populations (including geography, setting, endemic status and time period covered) and outcomes (key findings, limitations and conclusions) relevant to the review question were included. The draft form was tested on five records by one member of the review team and reviewed by the whole team. Data extraction was conducted manually using a piloted form in Covidence. Extracted records were checked by a second reviewer for completeness and consistency, and any discrepancies were resolved through discussion within the review team.

#### Data analysis and presentation

The studies included in the review were categorised according to expert opinion in a three-step process. First, papers were initially placed into three categories according to the main influence they identified: (1) climatic factors, (2) climatic factors and human behaviour and (3) human behaviour. Second, they were grouped based on the main outcome they assessed: (1) vector distribution and abundance, (2) other vector parameters, (3) YF infection and disease transmission. Third, they were categorised according to their conclusions on the risk of YF expansion: (1) no expansion or regression, (2) low likelihood of expansion and (3) high likelihood of expansion. The focus of non-global studies on endemic/non-endemic countries was also noted.

The studies were then collectively analysed using BoxAI, a generative artificial intelligence (genAI) tool, to extract the main trends (consequences and covariates) described in the studies. ‘Consequences’ were defined as effects resulting from either climatic factors or human behaviour on each of the three outcomes; ‘covariates’ were defined as variables used to characterise climatic factors or human behaviour. Details on the workflow and final prompts used are described in the online supplemental material protocol S3. BoxAI was used only to summarise information from completed extraction tables and to assist with the initial drafting of descriptive trend summaries. All final thematic categories, study allocations and interpretations were determined manually by the review team. For each consequence or covariate associated with vector distribution and abundance, other vector parameters or YF infections and disease transmission, a ‘median impact score’ was calculated based on the expert assessment to indicate the overall direction of reported findings across studies within each row (ie, no, low or high likelihood of expansion of YF). A numerical value was assigned to each study, where 0 = ‘no likelihood of expansion’, 1 = ‘low likelihood of expansion’ and 2 = ‘high likelihood of expansion’. For each consequence or covariate assessed, the median value across the respective included studies was then included as the ‘median impact score’. This score is intended as a guide and should not be interpreted as a formal measure of evidence strength or certainty.

## Results

### Literature search and characteristics of identified studies

**Figure 1:**
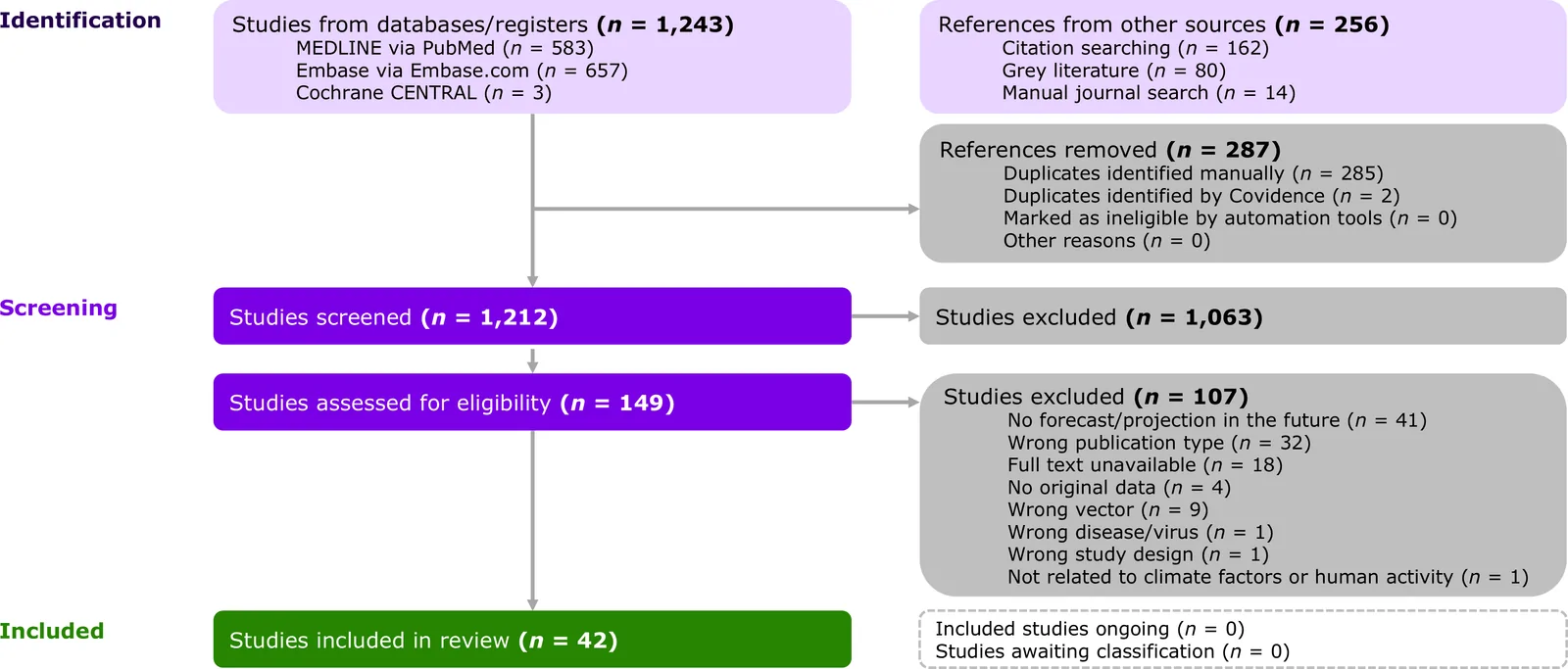
PRISMA flow diagram of the literature search and screening process. PRISMA, Preferred Reporting Items for Systematic Reviews and Meta-Analyses.

The results of the literature search are presented in a PRISMA-ScR flow diagram (figure 1).^31^ A total of 42 studies matched inclusion criteria and were included in the review, from an initial pool of 1243 from databases and registers, and 256 from other sources (figure 1).

Of the 42 studies, 81% (n=34/42) were modelling studies (online supplemental material table S1), with the remainder comprising observational studies (n=4/42, 9.5%) and studies employing mixed methods (n=4/42, 9.5%) (online supplemental material figure S1). None of the publications were older than 15 years (2009), with 67% (n=28/42) published between 2019 and 2024.

YF-endemic areas were the focus of 33% (n=14/42) of studies and non-endemic areas were the focus of 40% (n=17/42) of studies. Global populations were analysed in 26% (n=11/42) of studies. Of the non-global studies, only 10% (n=3/31) focused on Africa, while 35% focused on South America (n=11/31), and 19% on North America (n=6/31).

**Figure 2:**
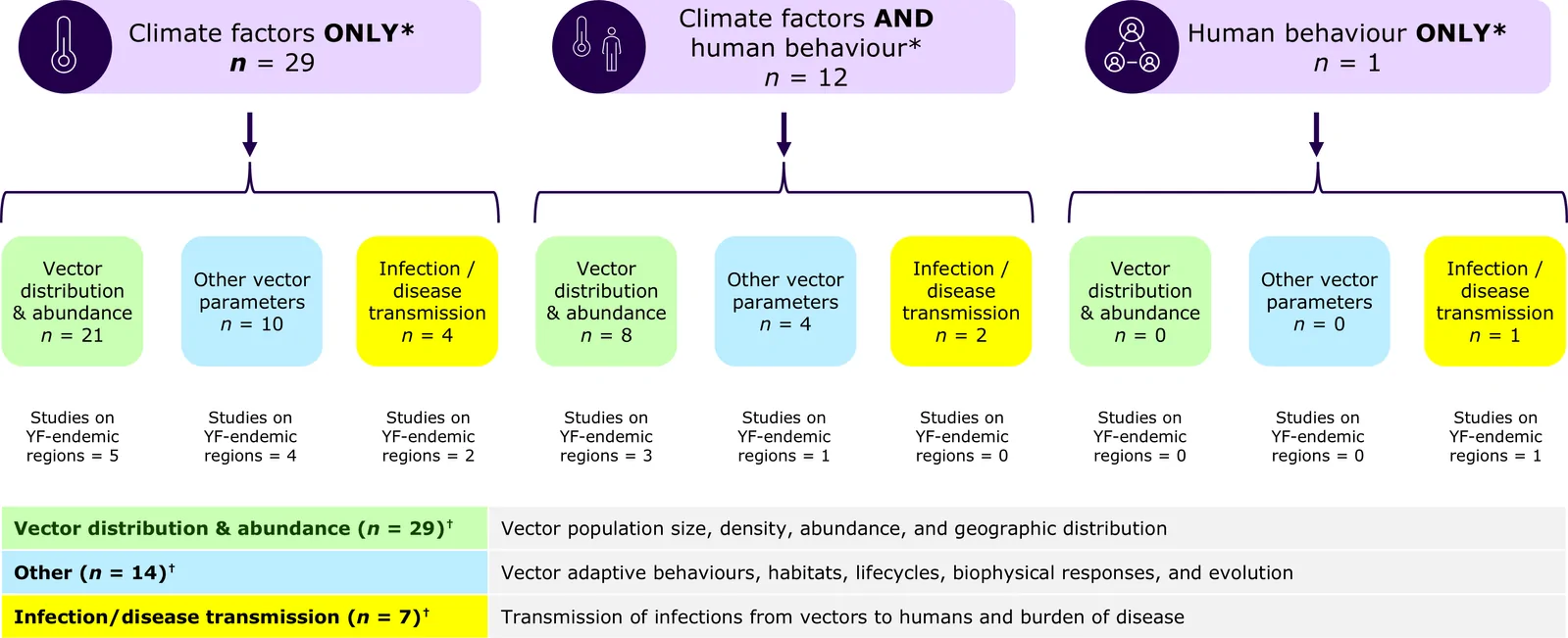
Classification of included studies. *n=42 as studies were classified as assessing either climate factors only, climate factors and human behaviour together, or human behaviour only. ^†^n>42 because some papers assessed more than one outcome, for example, vector distribution and abundance and other vector parameters. YF, yellow fever.

Approximately 69% (n=29/42) of the studies examined the effects of climate factors only, while 29% (n=12/42) examined the joint effects of climate and human behaviour and 2% (n=1/42) examined human behaviour only (figure 2). Some papers assessed more than one outcome across those three classifications: 29 studies considered vector distribution and abundance, 14 studies considered other vector parameters and 7 studies considered infection/disease transmission (figure 2).

### Prevalence of vector species in the included studies

Twelve species are currently recognised as YFV vectors, which are subdivided into urban (*Ae. aegypti*) and arboreal (various) species. Arboreal vectors are further subdivided into those found in the USA (*Sa. chloropterus, Hg. janthinomys, Hg. capricorni, Hg. leucocelaenus*) and in Africa (*Ae. vittatus, Ae. taylori, Ae. simpsoni, Ae. metallicus, Ae. leuteocephalus, Ae. furcifer, Ae. africanus*). Most attention in the literature dataset focused on *Ae. aegypti* (featured in n=39/42 publications), with no other YFV vector species occurring in more than 2 studies and 5 species (*Ae. furcifer, Ae. metallicus, Ae. simpsoni, Ae. taylori, Hg. capricorni*) being absent completely (online supplemental material figure S2).

There were seven publications that assessed the effects of climatic factors and/or human behaviour on diseases transmitted by YF vectors (YF, n=3/7; dengue fever, n=1/7). One dengue fever publication was included as it assessed the impact of climatic factors on the YF vector *Ae. aegypti*. Three of the publications did not focus on a specific vector-borne disease but did consider disease transmission and human exposure to YF vectors.

### Effect of climatic factors and human behaviour on vector distribution and abundance

In all, 29 studies (27 original research articles, 2 congress abstracts) considered vector distribution and abundance (figure 2).^12 25 33–59^ A complete list is in the online supplemental material (online supplemental table S2, S3). Of these, 25 were predictive modelling studies, 2 were observational/predictive modelling studies, 1 was an observational study and 1 was an entomological survey/predictive modelling study. The 29 studies collectively assessed 7 geographic regions (Global, Europe, Mediterranean, Southeast Asia, North Africa, North America. and South America), and 12 individual countries (Argentina, Australia, Brazil, Canada, China, Colombia, Ecuador, Iran, the autonomous region of Madeira, Mexico, Taiwan and the USA). In all, 21 studies considered vector distribution and abundance in the context of climatic factors only (figure 2).^33–53^ Eight studies considered vector distribution and abundance in the context of both climate factors and human behaviour (figure 2).^12 25 54–59^

All 29 studies considering vector distribution and abundance featured *Ae. aegypti*, with 52% (n=15/29) evaluating it exclusively. The main trends in these papers are summarised, along with the degree of variability in each trend and the number of supporting papers (table 2).

**Table 2:** Effects of climatic factors and human behaviour on vector distribution and abundance

	Trend	Variability	Number of studies evaluating this trend*	Median impact score†
**Consequences**	Vector range expansion Climate factors are likely to lead to a latitudinal expansion of suitable habitats for YF vectors, particularly in North America, Europe and parts of South America.	Variation of the specific regions identified and the extent (significant vs moderate) of the expansion.	19/29 studies.^12 33 35–38 41–44 46–48 52 54 56–59^	2
	Increased vector abundance Climate factors are projected to increase the overall abundance of YF vectors in certain areas due to favourable climatic conditions.	Variation in the predicted magnitude (substantial vs slight) of the increases.	8/29 studies.^34 36 37 45 47 48 53 78^	2
	Colonisation of urban areas Urban development and population density are significant factors driving the expansion of YF vector populations into new areas, especially urban settings where containers can harbour mosquito eggs.	Considerable variability in how different studies quantify this impact, with some emphasising rapid infestations in densely populated regions, while others note slower or less pronounced effects depending on local infrastructure and sanitation practices.	5/29 studies.^12 25 54 56 58^	2
	Increased distribution due to human activity The movement of people and changes in land use are expected to facilitate the spread of YF vectors into new areas.	Variability in the degree of risk assessed; some studies predict significant increases in vector presence due to high mobility and land use changes, whereas others suggest that the impact may be limited by other environmental factors.	4/29 studies.^12 25 54 58^	2
**Covariates**	Temperature‡ Critical for determining suitable habitats and affecting the distribution and abundance of YF vectors.	Strong consensus, though variability exists regarding the specific temperature thresholds that affect mosquito populations.	22/29 studies.^12 34 36–38 40–49 52 53 55–57 59 78^	2
	Precipitation Essential for vector dynamics, with an influence on habitat suitability and seasonal population fluctuations.	Moderate variability in how different geographic regions respond to changes in precipitation levels.	17/29 studies.^12 34 35 38 40–46 48 49 53 55 59 78^	2
	Land use Changes in land use, including urbanisation and agricultural practices, help create favourable conditions for YF vectors.	General agreement, but with variability in the types of land use changes considered impactful across different geographic contexts.	7/29 studies.^12 25 43 44 49 54 55^	2
	Human population density Particularly relevant in urban areas where breeding sites may be more abundant.	Variability in the extent to which population density is reckoned to affect vector dynamics in various locations.	3/29 studies.^12 25 42^	2

* Colour-coded according to the proportion of studies which support it. Dark blue indicates ≥66.6%, medium blue <66.6% and ≥33.3%, and light blue <33.3% of studies support the trend

† Median impact score was calculated using an ordinal scale whereby each included study in a row was assigned a numerical value based on an expert assessment of the likelihood of expansion according to the reported results; 0=no expansion or regression, 1=low likelihood of expansion, and 2=high likelihood of expansion. For each row, the median value of the included studies was then included as the median impact score.

‡ Including changes in the concentration of carbon dioxide and other greenhouse gases that can directly impact temperature.

YF, yellow fever.

The studies presented a clear consensus that vector (primarily *Ae. aegypti*) distribution is expected to increase with the colonisation of new (non-endemic) and urban areas. Human activities, principally population movements and land use changes, are projected to facilitate this range expansion, and the overall abundance of vectors is also forecast to increase. Temperature and precipitation are the key covariates for this expansion of vector distribution and abundance. Vector range expansion as a consequence and temperature and precipitation as covariates were evaluated in a high proportion of the included studies. All consequences and covariates assessed had a median impact score of 2, indicating high likelihood of expansion.

### Effects of climatic factors and human behaviour on other vector parameters

Fourteen studies (13 original research articles and 1 congress abstract) considered other vector parameters such as vector adaptive behaviours, habitat, life cycle, biophysical responses and evolution (figure 2).^34 37 47 49 52 53 56 59–65^ A full list is in the online supplemental material (online supplemental table S4, S5). Of these, 9 were predictive modelling studies, 4 were observational/predictive modelling studies and 1 was an observational study. The 14 studies collectively assessed 7 geographic regions (Global, central Amazon, Southeast Asia, Mediterranean, Africa, Caribbean and Latin America) and 5 individual countries (Australia, Brazil, Colombia, the autonomous region of Madeira and the USA).

Ten studies considered other vector parameters such as vector adaptive behaviours, habitat, life cycle, biophysical responses and evolution in the context of climatic factors only (figure 2).^34 37 47 49 52 53 60–63^ Four studies considered other vector parameters in the context of both climatic factors and human behaviour (figure 2).^56 59 64 65^

All the 14 studies assessed *Ae. aegypti,* 12 of them exclusively. There was little consensus in terms of the expected consequences of climatic factors and human behaviour on other vector parameters (table 3). More agreement between the studies was observed for the climatic and population-based covariates that were expected to affect vectors, with 75% (n=3/4) also being identified as factors impacting vector distribution and abundance (compare covariates in tables 3 and 2).

**Table 3:** Effects of climatic factors and human behaviour on other vector parameters

	Trend	Variability	Number of studies evaluating this trend*	Median impact score†
**Consequences**	Increased suitability for vector life cycle Climate change is predicted to create conditions favourable for the life cycles of *Ae. aegypti*.	General agreement.	3/14 studies.^47 60 63^	2
	Vector morphological changes Significant changes in the morphometry of *Ae. aegypti* eggs and embryos due to climatic factors, which may impact population density and arbovirus dynamics.	N/A.	1/14 study.^61^	1
	Ecological opportunism of vectors Vectors exhibit behavioural plasticity, such as moving to underground breeding sites in response to water scarcity exacerbated by extreme climatic events.	General agreement.	2/14 studies.^56 64^	1.5
	Increased biting of humans Rapid urbanisation might increase human-biting behaviour among vectors, driven by increased human population density.	N/A.	1/14 study.^65^	1
**Covariates**	Temperature‡ Influences various vector parameters such as life cycle, development rates, and morphological changes.	General agreement.	13/14 studies.^34 37 47 49 52 53 56 59–64^	1
	Precipitation Affects vector behaviour and habitat suitability.	Moderate agreement across studies.	6/14 studies.^34 49 52 59 60 65^	1
	Humidity Influences vector behaviour and survival.	Moderate; importance varies across different contexts within the studies.	3/14 studies.^56 61 62^	1
	Human population density Affects vector behaviour and adaptive strategies.	Moderate; emphasis varies depending on the study focus.	3/14 studies.^59 64 65^	1

* Colour-coded according to the proportion of studies which support it. Dark blue indicates ≥66.6%, medium blue <66.6% and ≥33.3%, and light blue <33.3% of studies support the trend.

† Median impact score was calculated using an ordinal scale whereby each included study in a row was assigned a numerical value based on an expert assessment of the likelihood of expansion according to the reported results; 0=no expansion or regression, 1=low likelihood of expansion, and 2=high likelihood of expansion. For each row, the median value of the included studies was then included as the median impact score.

‡ Including changes in the concentration of carbon dioxide and other greenhouse gases that can directly impact temperature.

Despite the lower consensus on the effects of climatic factors and human behaviour on other vector parameters, all of the trends identified in these studies (life cycle, development rates, morphological, ecological and behavioural changes) would be expected to facilitate YF spread. Temperature and precipitation were the most studied covariates influencing YF vectors. Most consequences and covariates assessed had a median impact score of 1, indicating low likelihood of expansion, with the exceptions of the ‘increased suitability for vector life cycle’ and ‘ecological opportunism of vectors’ consequences, which had median impact scores of 2 and 1.5, respectively.

### Effects of climatic factors and human behaviour on YF infections and disease transmission

Six studies considered impacts on YF infection and disease transmission (figure 2).^17 51 66–69^ All 6 studies were original research articles, including 5 predictive modelling studies and 1 observational/predictive modelling study. The studies assessed 3 geographic regions (Global, Africa and Asia-Pacific) and 2 individual countries (Brazil and the USA).

Four studies considered the impact of climatic factors on YF infection and disease transmission (figure 2).^17 51 66 67^ Two studies considered YF infection and disease transmission in the context of both climatic factors and human behaviour (figure 2).^68 69^ In addition, 1 study considered the impact of human behaviour only on YF infection and disease transmission.^70^ A complete list of studies in this category is provided in online supplementary material (online supplemental table S6–S8).

The studies were heterogeneous in focus, with several of the identified trends deriving from one study only (table 4).

**Table 4:** Effects of climatic factors and human behaviour on YF infection and disease transmission

	Trend	Variability	Number of studies evaluating this trend*	Median impact score†
**Consequences**	Higher transmission risk Due to increased suitability for mosquito vectors in various regions from the effects of climate change.	Variability in the specific regions identified and the degree of increased risk projected.	4/7 studies.^17 66–68^	1
	Expansion of transmission areas Projected increases in the infections and potential expansion of YF transmission areas due to climate change, especially outside West Africa.	Variability regarding which areas will be most affected and how significant these changes will be.	3/7 studies.^17 67 69^	2
	Decreases in cases due to temperature limits Rising temperatures could exceed suitable ranges for mosquito survival, potentially resulting in decreased YF cases.	Contrasts with studies projecting higher transmission risk.	1/7 study.^51^	0
	Increased exposure due to population growth and urbanisation Expected to increase exposure to YFV, particularly in emerging economies.	Variability in the extent of the impact across different regions, especially between emerging and industrialised economies.	3/7 studies.^17 68 69^	1
	Increased risk of YF infection and transmission due to land use changes Changes in land use and habitat fragmentation increase the risk of YF occurrences, indicating a transition period where sylvatic spillover is more likely.	N/A.	1/7 study.^70^	1
	Increased exposure of vulnerable groups Socioeconomic and demographic factors influence vulnerability and exposure to YFV across different regions and populations.	N/A.	1/7 study.^69^	2
**Covariates**	Temperature‡ A critical covariate influencing mosquito survival and thereby YFV transmission dynamics.	Variation in specific temperature ranges considered optimal for different mosquito species and how these temperatures interact with other factors.	6/7 studies.^17 51 66–69^	1
	Precipitation Affects mosquito breeding habitats and consequently YF transmission.	Variation in the thresholds of rainfall that promote or inhibit mosquito populations.	3/7 studies.^51 66 68^	1
	Human population density Higher human population densities increase exposure to YFV, and consequently YF transmission.	Variation in quantification of this relationship and its implications for different geographic regions.	3/7 studies.^17 68 69^	1
	Land use and habitat fragmentation Can alter ecological dynamics and affect YF transmission risk.	N/A.	1/7 study.^70^	1
	Wind speed and cloud cover Potential effects on mosquito dispersal and environmental conditions conducive to transmission.	N/A.	1/7 study.^67^	2

* Colour-coded according to the proportion of studies which support it. Dark blue indicates ≥66.6%, medium blue <66.6% and ≥33.3%, and light blue <33.3% of studies support the trend.

† Median impact score was calculated using an ordinal scale whereby each included study in a row was assigned a numerical value based on an expert assessment of the likelihood of expansion according to the reported results; 0=no expansion or regression, 1=low likelihood of expansion, and 2=high likelihood of expansion. For each row, the median value of the included studies was then included as the median impact score.

‡ Including changes in the concentration of carbon dioxide and other greenhouse gases that can directly impact temperature.

YF, yellow fever; YFV, yellow fever virus.

Although several studies projected higher transmission risk or broader exposure under some climate and population scenarios, the evidence base for direct YF infection and transmission outcomes was limited and heterogeneous. Temperature and precipitation were again identified as the main covariates, with human population density also a key variable. The ‘higher transmission risk’, ‘expansion of transmission areas’ and ‘increased exposure due to population growth and urbanisation’ consequences and temperature, precipitation and human population density covariates were evaluated in a high proportion of the included studies. The majority of consequences and covariates had a median impact score of 1, although the ‘expansion of transmission areas’ and ‘increased exposure of vulnerable groups’ consequences and the wind speed and cloud cover covariate had a higher median impact score of 2, while the ‘decreases in cases due to temperature limits’ consequence had a lower median impact score of 0.

## Discussion

YF is an acute viral disease spread by several mosquito species. Climatic factors and human behaviour have the potential to alter the distribution of these vectors, with a concomitant impact on the geographic range of the YFV.^10 71 72^ Here, 42 studies were identified that anticipated the role of climatic factors and human behaviour in the future spread of YF in 3 main areas: (1) vector distribution and abundance, (2) other vector parameters, (3) YFV infection and disease transmission. A genAI-based approach with human oversight was used to extract consensus trends. These trends are necessarily speculative, given the variability in the studies, the subjective element in data extraction, and the absence of a systematic quantitative analysis. Nonetheless, several common themes emerged.

The studies projected a potential range expansion of *Ae. aegypti* into new regions, particularly in North America, Europe and parts of Asia. This projected expansion is expected to be accompanied by increased vector abundance in endemic and non-endemic areas. The vector range expansion and abundance increase are expected to be driven primarily by rising temperatures and changing precipitation patterns. There was evidence that vector life cycles and population densities may be affected, with projections of increased seasonal variation in vector population densities and adaptations in breeding behaviours. These alterations were principally linked to urbanisation and environmental changes. Several studies projected increased human exposure to *Aedes*-borne viruses in densely populated areas. However, direct evidence on future YF transmission was more limited and should not be inferred automatically from projected increases in vector suitability or abundance.

Several significant knowledge gaps were identified. Despite a broad definition of human behaviour and the inclusion of search terms relating to immunisation, prevention and vector control, few studies addressed these topics in the context of future trends. There was a lack of publications on the role of YF vectors other than *Ae. aegypti* in the future spread of the disease*,* despite the importance of these species for YFV transmission to humans. ^73 74^All recent YF outbreaks in South America have been linked with sylvatic transmission involving *Haemagogus* and *Sabethes* mosquitoes rather than *Ae. aegypti*. In fact, the last documented isolation of YFV from *Ae. aegypti* in this region dates from 1942.^75^ Recently, *Ae. scapularis* and *Psorophora* spp (especially *Psorophora* ferox), as well as *Aedes opok* and members of the *Aedes simpsoni* complex (notably *Ae. bromeliae*) have all attracted attention as potential YFV vectors in the Americas, while YFV-infected *Ae. albopictus* have been observed in the field, making it a possible bridge vector.^74 76^ These recent outbreaks underline the need to include non-*Ae. aegypti* species in future projections of YF spread.

Sylvatic environments remain important reservoirs and drivers that may ultimately influence urban epidemics across the globe. Most recent YF outbreaks are thought to have originated from the movement of YFV-infected humans or NHPs, resulting in infection of local mosquito populations and facilitation of transmission at the local level. In parallel, human residential areas offer more artificial breeding sites and more sources of blood for mosquitoes, which increases local mosquito population densities. During the recent YF epidemic in Brazil, increasing surveillance of NHPs was considered a priority. The reactivity and the lack of cross-protective immunity in NHPs are important considerations for future research.

Given that Africa bears the highest burden of YF, the relative lack of studies focusing on the role of climate variability and climate change in the future spread of YF in Africa represents a major gap in the available data. This is especially notable given the absence of any geographical restrictions in the search criteria, and addressing it should be a priority for future research. African NHPs do not die from YF infections, and studies of NHPs and YF in Africa have been limited to occasional serological studies. Paradoxically, despite increasing vaccine coverage, YF outbreaks continue to occur in Africa, suggesting that the variables governing YF dynamics in this region are not fully understood.^23^ There is an urgent need for a better understanding of YF epidemiology in Africa and the interplay between humans and NHPs in transmission and persistence of the disease. Why YF is absent from Asia and the Pacific region despite the ubiquity of competent *Ae. aegypti* vectors and favourable climate conditions is an enduring puzzle which may have implications for YF control in other parts of the world.^27–29 77^

### Limitations

The study has several limitations. While scoping reviews map the breadth and nature of existing literature to identify key concepts and research gaps, they are inherently limited by the lack of assessment of methodological rigour and bias. Moreover, it is very likely that some relevant articles were not captured in the dataset, and congress abstracts from the last 2 years only were included in the review. A structured post-cut-off PubMed check suggested continued publication activity after September 2024, with ~150 new records fitting the search criteria as of June 2026. Based on the proportions of identified publications from each source relative to PubMed in the original search, the potential number of new studies was estimated to amount to ~280 for title/abstract screening, ~40 for full text screening and only ~10 for addition to the analysis. However, the actual extent and effect of any potential additional records on the mapped themes cannot be determined without full screening and extraction. Additionally, much of the included literature addressed vector suitability, abundance or vector-related parameters rather than direct YF transmission outcomes, limiting the extent to which epidemic risk can be inferred. As the literature search strategy focused on future trends (online supplemental material protocol S1), studies dealing with contemporary vaccine policy, healthcare systems and decision-making were not captured. Immunisation was included in the human behaviour category of the protocol to cover the effect of vaccination coverage, but the impact of healthcare system activities is an important dimension that was not included in the study design. Some of the obtained datasets in the assessment sub-categories were small, and the identified trends and corresponding levels of risk and evidence should be treated with caution. The analysis of the studies was ultimately based on expert opinion, and therefore all conclusions have a degree of subjectivity. The predominance of modelling studies in the dataset reflects the focus of this scoping review on the future spread of YF rather than retrospective analysis, but also underscores the urgent need for complementary epidemiological and field studies. The original data for many of the studies were not available, making verification of modelling inferences difficult. Most included modelling studies were ecological niche, species-distribution or process-based mechanistic models, with temperature and precipitation the dominant climatic inputs. A smaller subset also incorporated land use, population density, mobility or emissions scenarios. This concentration of modelling assumptions helps explain the recurrent emphasis on climatic suitability and vector range expansion across the literature.

## Conclusion

The interactions between climatic, behavioural, ecological and health system factors must be viewed together to anticipate the future spread of YF. In this context, the studies collected in this scoping review specifically consider the interplay between climatic factors and human behaviour. The mapped literature suggests that climate change and human-driven environmental change may increase the suitability and distribution of major YF vectors, especially *Ae. aegypti*. Evidence linking these projections to future YF transmission and epidemic risk is more limited, highlighting the need for studies that integrate vector, sylvatic, vaccination and health-system dynamics. Assessing the factors that influence YF transmission will help guide future research, inform public health policy and support preventive action. Despite the importance of non-*Aedes* species in recent YF outbreaks and the central importance of Africa, there is a clear gap in the current literature considering how these factors will be impacted by climate change and human behaviour in the context of the future spread of YF. Addressing these gaps will be important for anticipating effective public health strategies. Prioritising an understanding of these complex interactions will assist mitigation of YF expansion and will help to improve preparation for and response to YF epidemics in the future.

## Supplementary material

10.1136/bmjopen-2025-115595 online supplemental file 1

## Data Availability

Data are available upon reasonable request.
