# Supplementary material for: Projecting the future spread of yellow fever vectors and implications for epidemic risk: a scoping review of climate change and human behaviour
Source: BMJ Open. 2026 Sep 21;16(9):e115595. doi: 10.1136/bmjopen-2025-115595 (PMC13599982; doi:10.1136/bmjopen-2025-115595)
Supplement: Supplementary file 1 [file bmjopen-2025-115595.reviewer_comments.pdf]

PEER REVIEW HISTORY

BMJ Open publishes all reviews undertaken for accepted manuscripts. Reviewers are asked to complete a checklist review form and are provided with free text boxes to elaborate on their assessment. These free text comments are reproduced below.

ARTICLE DETAILS

Title (Provisional)

PROJECTING THE FUTURE SPREAD OF YELLOW FEVER VECTORS AND IMPLICATIONS FOR EPIDEMIC RISK: A SCOPING REVIEW OF CLIMATE CHANGE AND HUMAN BEHAVIOUR

Authors

Parreiras Gonçalves, Andreza; Failloux, Anna-Bella; Jelinek, Tomas; LaBeaud, Angelle Desiree; Langevin, Edith; Ooi, Eng Eong; Thollot, Yaël; Tomori, Oyewale; Vasilakis, Nikos; Lowe, Rachel; Colón-González, Felipe J

VERSION 1 - REVIEW

**Reviewer**	**1**
**Name**	**Yeh-Li, Ho**
**Affiliation**	**Hospital das Clínicas da Faculdade de Medicina USP, Division of Infectious and Parasitic Diseases**
**Date**	**09-Feb-2026**
**COI**	**None**

The authors conducted a literature review using the PRISMA-ScR strategy to evaluate the impact of climate change on the future risk of yellow fever transmission worldwide. The review framework was based on three main pillars: climate change, the vector, and human behavioral factors. The topic is highly relevant and timely, particularly considering that yellow fever is an immunopreventable disease with high lethality.

The review is extensive and presents findings of great importance for this arboviral disease. However, the manuscript is excessively long in some sections, with occasional repetition of concepts within the same paragraph.

**Reviewer**	**2**
**Name**	**Gaythorpe, Katy**
**Affiliation**	**Imperial College London Faculty of Medicine, School of Public Health, Department of Infectious Disease Epidemiology**

Date 25-Mar-2026

COI I report speaker fees from Sanofi for a conference presentation in May 2023.

This is a timely and interesting topic for review- particularly examining the impact of climate change alongside human behaviour. The review methods are appropriate in terms of thorough search strategy. However, to address the strength of evidence, it is necessary to compare study quality. Further, as a large proportion of the included studies were modelling focused, a more granular examination of the model approach/type and input data used would help to contextualise the evidence collated. The presentation of the results in the main text could be clearer- perhaps colour coded by the proportion of studies supporting each hypothesis (and both the strength of the association as well as the strength of the study itself). Finally, the inclusion of human behaviour is welcome but this largely fell into population distribution, some human movement and vaccination coverage- where there any studies that examined decision making?

Some minor comments include:

- avoid the term 'confined' in the abstract referring to geographic distribution as you make the point that the geographic distribution could change.

- some parts of the introduction do not flow narratively e.g. the final paragraph of page 7 reads more like a list of statements.

- on page 9, a vaccination threshold is mentioned below which outbreaks can occur. However, this sentence is discussing spillover which only requires coverage <100% rather than a herd-immunity type threshold.

- on page 9, "such outbreaks are often centred on unvaccinated individuals" I would question the use of often here- the majority of outbreaks occur in unvaccinated individuals.

- the statement of the aim on page 10 is disparate from the two identified information gaps mentioned in the previous paragraph- are they linked to the aims of the study?

**Reviewer**	**3**
**Name**	**Arcos Gonzalez, Pedro**
**Affiliation**	**Universidad de Oviedo, Medicine. Public Health**
**Date**	**08-Apr-2026**
**COI**	**None**

Since it is not a systematic review, but only a scoping reviews, it is not possible to reach relevant conclusions. On the other hand, many of the supposed conclusions were already well known beforehand.

VERSION 1 - AUTHOR RESPONSE

Reviewer 1

The authors conducted a literature review using the PRISMA-ScR strategy to evaluate the impact of climate change on the future risk of yellow fever transmission worldwide. The review framework was based on three main pillars: climate change, the vector, and human behavioral factors. The topic is highly relevant and timely, particularly considering that yellow fever is an immunopreventable disease with high lethality. The review is extensive and presents findings of great importance for this arboviral disease.	We thank the reviewer for their positive assessment.
However, the manuscript is excessively long in some sections, with occasional repetition of concepts within the same paragraph.	We have trimmed the manuscript text throughout, with particular attention being paid to removal of repetitive elements. In particular, we have removed or reworded text in the introduction (lines 162 – 165,176 – 178, 181 – 185, 187, 195 – 197, 201, 204 – 205, 208 – 211, 222 – 227), results (lines 404 – 410, 411 – 418, 456 – 463, 464 – 470, 505 – 509, 510 – 513,) and discussion (lines 556 – 558, 570 – 573, 577, 580 – 583, 592, 608 – 610). We hope this sufficiently addresses the reviewer's point.

Reviewer 2

This is a timely and interesting topic for review- particularly examining the impact of climate change alongside human behaviour. The review methods are appropriate in terms of thorough search strategy.	We appreciate the reviewer's assessment of the timeliness and relevance of this work, and their positive assessment of the methods we used.
However, to address the strength of evidence, it is necessary to compare study quality.	We agree that contextualising the strength of the evidence is important. Although we did not undertake formal critical appraisal, in keeping with the scoping-review design, we have strengthened the manuscript by clarifying the predominance of modelling studies, expanding the limitations section, and adding a supplementary table (Table S1 – see also

	response below) that summarises model approaches, model types, and input data.
Further, as a large proportion of the included studies were modelling focused, a more granular examination of the model approach/type and input data used would help to contextualise the evidence collated.	We have added a new supplementary table (Table S1) summarising model approach, model type, and input data for all included modelling studies, and we have highlighted the predominance of ecological niche/species-distribution and process-based models and their reliance on climatic inputs at the end of the limitations section in the Discussion.
The presentation of the results in the main text could be clearer- perhaps colour coded by the proportion of studies supporting each hypothesis (and both the strength of the association as well as the strength of the study itself).	Thank you for your suggestion; we have implemented it as follows:<br>• The "support" column in Tables 1–3 (now Tables 2–4) has been been relabelled as "Number of studies evaluating this trend" and updated with a 3-point colour gradient to reflect the proportion of studies supporting each identified trend.<br>• We have introduced a descriptive ordinal summary ('median impact score', scaled 0/1/2) to indicate the direction of reported findings across studies. We recognise that this does not constitute a formal assessment of evidence strength or certainty, and we have clarified this in the manuscript. A numerical value is assigned to each study based on the "no/low/high likelihood of expansion" classifications in Tables S2-S8, whereby 0 = "no likelihood of expansion", 1 = "low likelihood of expansion" and 2 = "high likelihood of expansion". For each row, the median value of the included studies is then included as the "median impact score". These scores are discussed in the text on pages 17 & 18 (lines 343 – 352), 23 (lines 442 – 443), 27 (lines 493 – 496) and 30 & 31 (lines 541–545).
Finally, the inclusion of human behaviour is welcome but this largely fell into population distribution, some human movement and vaccination coverage-	We agree that examination of the impact of decision making on this topic would be interesting. However, no such studies were identified which fit the inclusion/exclusion criteria for this scoping review. It is possible that the requirement to include data related to future

where there any studies that examined decision making?	trends of YF epidemiology or vector evolution excluded any studies focused on the impact of decision making, as this is more difficult to model accurately than effects due to other parameters. We have added this point to the Limitations section of the manuscript on page 34, line 634.<br><br>We have explicitly defined human behaviour in the context of the present analysis as referring to “human-driven demographic, mobility, land-use, vaccination, and vector-control factors that may interact with environmental change and alter vector range, human exposure, or transmission dynamics” in the Introduction section of the revised manuscript (page 11, lines 212–215).
**Minor comments:**	
- avoid the term 'confined' in the abstract referring to geographic distribution as you make the point that the geographic distribution could change.	We thank you for your suggestion; this has been implemented as requested (pages 3 and 8).
- some parts of the introduction do not flow narratively e.g. the final paragraph of page 7 reads more like a list of statements.	We have revised the final two paragraphs of the introduction to improve transitions between the discussion of vaccination, ecological uncertainty, and the study aim.
- on page 9, a vaccination threshold is mentioned below which outbreaks can occur. However, this sentence is discussing spillover which only requires coverage <100% rather than a herd-immunity type threshold.	Thank you for highlighting this issue; we have amended the statement as shown (page 10, lines 201–206):<br><br>Furthermore, sustained urban transmission is unlikely t<br>of robust YFV vaccination coverage.[22 23] Nevertheless, h<br>policies can vary greatly from one region to another, and<br>remain unvaccinated, spillover events can still lead to o<br>~~in urban settings drops below the threshold required to~~<br>~~(~~such as the ~~one~~ outbreak in Brazil in 2016–2018~~) do occ~~
- on page 9, "such outbreaks are often centred on unvaccinated individuals" I would question the use of often here- the majority of outbreaks occur in unvaccinated individuals.	We have amended the statement as shown (page 10, lines 206–208).

	(such as the ~~one~~ outbreak in Brazil in 2016–2018) ~~do oc~~<br>among unvaccinated individuals, who account for the r<br>cases. ~~Such outbreaks are often centred on unvaccinat~~
- the statement of the aim on page 10 is disparate from the two identified information gaps mentioned in the previous paragraph- are they linked to the aims of the study?	We have clarified the text of the paragraph preceding the statement of the aim as shown (page 12, lines 228 – 232):<br>viruses has been suggested as a possible explanation<br>highlight the difficulty of predicting YF spread from vec<br>alone, and suggest that future work must better ~~integra~~<br>ecological, and human behavioural drivers~~factors mus~~<br>integrated framework.

Reviewer 3

Since it is not a systematic review, but only a scoping reviews, it is not possible to reach relevant conclusions. On the other hand, many of the supposed conclusions were already well known beforehand.	We appreciate this concern and agree that a scoping review cannot provide the same type of inferential conclusion as a systematic review with formal critical appraisal. We have therefore revised the manuscript to present the findings more clearly as a mapping of the available literature, highlighting where evidence converges and where important gaps remain. The value of the review lies in bringing together a fragmented body of projection-focused literature on climate, vector dynamics, and human-related drivers, rather than in claiming definitive causal conclusions.

VERSION 2 - REVIEW

Reviewer 2

Name Gaythorpe, Katy

Affiliation Imperial College London Faculty of Medicine, School of Public Health, Department of Infectious Disease Epidemiology

Date 13-Jul-2026

COI

The manuscript is significantly improved in the revision.
