# Supplementary material for: Projecting the future spread of yellow fever vectors and implications for epidemic risk: a scoping review of climate change and human behaviour
Source: BMJ Open. 2026 Sep 21;16(9):e115595. doi: 10.1136/bmjopen-2025-115595 (PMC13599982; doi:10.1136/bmjopen-2025-115595)
Supplement: Supplementary file 2 [file bmjopen-16-9-s001.docx]

**Supplementary files**

**Table of Contents**

[Checklist S1 PRISMA ScR checklist	2](#_Toc233047896)

[Protocol S1 Literature search strategies	4](#_Toc233047897)

[PubMed	4](#_Toc233047898)

[Embase	5](#_Toc233047899)

[Cochrane CENTRAL	6](#_Toc233047900)

[Protocol S2 Data extraction form	8](#_Toc233047901)

[Protocol S3 GenAI usage	9](#_Toc233047902)

[Table S1 Characteristics of the included modelling studies	10](#_Toc233047903)

[Figure S1 Characteristics of the included studies	15](#_Toc233047904)

[Figure S2 Prevalence of yellow fever vector species in the literature dataset	16](#_Toc233047905)

[Table S2 Studies on the effects of climatic factors on vector distribution and abundance	17](#_Toc233047906)

[Table S3 Studies on the effects of climatic factors and human behaviour on vector distribution and abundance	21](#_Toc233047907)

[Table S4 Studies on the effects of climatic factors on other vector parameters	23](#_Toc233047908)

[Table S5 Studies on the effects of climatic factors and human behaviour on other vector parameters	26](#_Toc233047909)

[Table S6 Studies on the effects of climatic factors on YF infection and disease transmission	27](#_Toc233047910)

[Table S7 Studies on the effects of climatic factors and human behaviour on YF infection and disease transmission	29](#_Toc233047911)

[Table S8 Studies on the effects of human behaviour on YF infection and disease transmission	30](#_Toc233047912)

# Checklist S1 PRISMA ScR checklist

| SECTION | ITEM | PRISMA-ScR CHECKLIST ITEM | REPORTED ON PAGE # |
| --- | --- | --- | --- |
| TITLE | | | |
| Title | 1 | Identify the report as a scoping review. | Page 1 |
| ABSTRACT | | | |
| Structured summary | 2 | Provide a structured summary that includes (as applicable): background, objectives, eligibility criteria, sources of evidence, charting methods, results, and conclusions that relate to the review questions and objectives. | Page 3-5. Abstract contains background, objectives, eligibility criteria, sources of evidence, charting methods, results, and conclusions |
| INTRODUCTION | | | |
| Rationale | 3 | Describe the rationale for the review in the context of what is already known. Explain why the review questions/objectives lend themselves to a scoping review approach. | Page 6-9 |
| Objectives | 4 | Provide an explicit statement of the questions and objectives being addressed with reference to their key elements (e.g., population or participants, concepts, and context) or other relevant key elements used to conceptualize the review questions and/or objectives. | Page 9 |
| METHODS | | | |
| Protocol and registration | 5 | Indicate whether a review protocol exists; state if and where it can be accessed (e.g., a Web address); and if available, provide registration information, including the registration number. | Page 10 - 15 |
| Eligibility criteria | 6 | Specify characteristics of the sources of evidence used as eligibility criteria (e.g., years considered, language, and publication status), and provide a rationale. | Page 10 - 15 |
| Information sources* | 7 | Describe all information sources in the search (e.g., databases with dates of coverage and contact with authors to identify additional sources), as well as the date the most recent search was executed. | Page 10 - 15 |
| Search | 8 | Present the full electronic search strategy for at least 1 database, including any limits used, such that it could be repeated. | Protocol S1 |
| Selection of sources of evidence† | 9 | State the process for selecting sources of evidence (i.e., screening and eligibility) included in the scoping review. | Page 10 - 15 |
| Data charting process‡ | 10 | Describe the methods of charting data from the included sources of evidence (e.g., calibrated forms or forms that have been tested by the team before their use, and whether data charting was done independently or in duplicate) and any processes for obtaining and confirming data from investigators. | Page 10 - 15, Protocol S2 |
| Data items | 11 | List and define all variables for which data were sought and any assumptions and simplifications made. | Page 10 - 15, Protocol S2 |
| Critical appraisal of individual sources of evidence§ | 12 | If done, provide a rationale for conducting a critical appraisal of included sources of evidence; describe the methods used and how this information was used in any data synthesis (if appropriate). | This was not  conducted for  this study |
| Synthesis of results | 13 | Describe the methods of handling and summarizing the data that were charted. | Page 10 - 15 |
| RESULTS | | | |
| Selection of sources of evidence | 14 | Give numbers of sources of evidence screened, assessed for eligibility, and included in the review, with reasons for exclusions at each stage, ideally using a flow diagram. | Pages 15-26 |
| Characteristics of sources of evidence | 15 | For each source of evidence, present characteristics for which data were charted and provide the citations. | Tables S1–S8 |
| Critical appraisal within sources of evidence | 16 | If done, present data on critical appraisal of included sources of evidence (see item 12). | This was not  conducted for  this study |
| Results of individual sources of evidence | 17 | For each included source of evidence, present the relevant data that were charted that relate to the review questions and objectives. | Pages 15-26 |
| Synthesis of results | 18 | Summarize and/or present the charting results as they relate to the review questions and objectives. | Pages 15-26 |
| DISCUSSION | | | |
| Summary of evidence | 19 | Summarize the main results (including an overview of concepts, themes, and types of evidence available), link to the review questions and objectives, and consider the relevance to key groups. | Page 26 -29 |
| Limitations | 20 | Discuss the limitations of the scoping review process. | Page 29 - 30 |
| Conclusions | 21 | Provide a general interpretation of the results with respect to the review questions and objectives, as well as potential implications and/or next steps. | Page 30 - 31 |
| FUNDING | | | |
| Funding | 22 | Describe sources of funding for the included sources of evidence, as well as sources of funding for the scoping review. Describe the role of the funders of the scoping review. | Page 33 |

*Where sources of evidence (see second footnote) are compiled from, such as bibliographic databases, social media platforms, and Web sites. †A more inclusive/heterogeneous term used to account for the different types of evidence or data sources (e.g., quantitative and/or qualitative research, expert opinion, and policy documents) that may be eligible in a scoping review as opposed to only studies. This is not to be confused with information sources (see first footnote). ‡The frameworks by Arksey and O’Malley (Arksey H and O’Malley L. Scoping studies: towards a methodological framework. *International Journal of Social Research Methodology* 2005;8(1):19–32), Levac and colleagues (Levac D, Colquhoun H and O’Brien KK. Scoping studies: advancing the methodology. *Implement Sci* 2010;5(69)) and the JBI guidance (Peters MJD, Marnie C, Tricco AC et al. Updated methodological guidance for the conduct of scoping reviews. *JBI Evid Synth.* 2020;18(10):2119–2126) refer to the process of data extraction in a scoping review as data charting. §The process of systematically examining research evidence to assess its validity, results, and relevance before using it to inform a decision. This term is used for items 12 and 19 instead of "risk of bias" (which is more applicable to systematic reviews of interventions) to include and acknowledge the various sources of evidence that may be used in a scoping review (e.g., quantitative and/or qualitative research, expert opinion, and policy document).

JBI, Joanna Briggs Institute; PRISMA-ScR, Preferred Reporting Items for Systematic reviews and Meta-Analyses extension for Scoping Reviews.

From: Tricco AC, Lillie E, Zarin W, O'Brien KK, Colquhoun H, Levac D, et al. PRISMA Extension for Scoping Reviews (PRISMAScR): Checklist and Explanation. Ann Intern Med. 2018;169:467–473. doi: 10.7326/M18-0850.

| **Search number** | **Query** |
| --- | --- |
| 1 | "Yellow Fever"[MAJR] OR "yellow fever"[ti] OR yellow-fever[ti] |
| 2 | "Aedes"[MAJR] OR Aedes[ti] OR Haemagogus[ti] OR Sabethes[ti] |
| 3 | "Mosquito Vectors"[MAJR] OR (("Culicidae"[MAJR] OR "culicidae"[ti] OR "mosquito"[ti] OR "mosquitoes"[ti]) AND disease*[ti]) OR "mosquito borne disease*"[ti] OR "mosquito-borne disease*"[ti] OR "vector borne disease*"[ti] |
| 4 | #1 OR #2 OR #3 |
| 5 | "future distribution"[ti:~3] OR "future spread"[ti:~3] OR “projected distribution*”[ti] |
| 6 | #4 AND #5 |
| 7 | future[ti] OR predict*[ti] OR forecast*[ti] OR evolution[ti] OR evolv*[ti] OR expansion[ti] OR expand*[ti] OR model*[ti] OR simulat*[ti] OR project*[ti] OR redistribut*[ti] OR "re-emerg*"[ti] OR chang*[ti] |
| 8 | #4 AND #7 |
| 9 | "Meteorological Concepts"[MAJR] OR "meteorological factor*"[ti] OR "Climate"[MAJR] OR "Climatic Processes"[Majr] OR "Climate Change"[MAJR] OR "climate change*"[ti] OR "climate alteration"[ti:~2] OR "climate alterations"[ti:~2] OR "climate projection*"[ti] OR climate[ti] OR climatic[ti] OR "climate variability"[ti:~2] OR "climate variation"[ti:~2] OR "climate crisis"[ti] OR "climate emergency"[ti] OR "climatic factor*"[ti] OR "climatic driver*"[ti] OR "anthropogenic climate change"[ti] OR "Weather"[MAJR] OR "weather alteration"[ti:~2] OR "weather alterations"[ti:~2] OR "weather pattern"[ti:~2] OR "weather patterns"[ti:~2] OR "Extreme Weather"[MAJR] OR "extreme weather"[ti] OR "Air Movements"[MAJR] OR "El Nino-Southern Oscillation"[MAJR] OR "ENSO"[ti] OR "el nino"[ti] OR "la nina"[ti] OR "Temperature"[MAJR] OR temperature*[ti] OR "Humidity"[MAJR] OR humidity[ti] OR Precipitation[ti] OR "Rain"[Majr] OR rain*[ti] OR "Droughts"[MAJR] OR drought*[ti] OR "Floods"[MAJR] OR flood*[ti] OR "Global Warming"[MAJR] OR "global warming"[ti] OR "Greenhouse Effect"[MAJR] OR "greenhouse effect"[ti] OR "carbon emissions"[ti] |
| 10 | "Conservation of Natural Resources"[MAJR] OR "exploitation natural resources"[ti:~1] OR deforest*[ti] OR "forest conversion"[ti:~2] OR desertification[ti] OR "land use change"[ti:~2] OR "land use"[ti] OR "Agriculture"[MAJR] OR farming[ti] OR "agricultural development"[ti] OR "land cover"[ti:~2] OR "environmental degradation"[ti] OR "environment degradation"[ti:~2] OR "forest fragmentation"[ti:~2] OR "Zoonoses"[MAJR] OR spillover[ti] OR "Urbanization"[MAJR] OR "urbani*ation"[ti] OR "urban expansion"[ti] OR "Socioeconomic Factors"[MAJR] OR "Socioeconomic Disparities in Health"[MAJR] OR socioeconomic[ti] OR "Population Forecast"[MAJR] OR "population projection*"[ti] OR "population growth estimat*"[ti] OR "Population Growth"[MAJR] OR "population growth"[ti] OR "Population Dynamics"[MAJR] OR "Rural Urban Migration*"[ti] OR "Human Migration"[MAJR] OR "human movement"[ti:~1] OR "movement of humans"[ti:~0] OR "human mobility"[ti] OR "human migration"[ti] OR "migration of humans"[ti:~0] OR "Health Transition"[MAJR] OR "Travel"[MAJR] OR travel[ti] OR trade[ti] OR "trade route*"[ti] OR "Behavior"[MAJR] OR "human behavio*r*"[ti] OR "human activit*"[ti] OR "Mosquito Control"[MAJR] OR "mosquito* control*"[ti] OR "mosquito control"[ti:~2] OR "mosquitoes control"[ti:~2] OR "vector* control*"[ti] OR "vector control"[ti:~2] OR "vectors control"[ti:~2] OR "Immunization"[MAJR] OR "immuni*ation"[ti] OR "Vaccination"[MAJR] OR "vaccin*"[ti] OR prevent*[ti] |
| 11 | #8 AND #9 |
| 12 | #8 AND #10 |
| 13 | #6 OR #11 OR #12 |

# Protocol S1 Literature search strategies

## PubMed

## Embase

| **No.** | **Query** |
| --- | --- |
| 1 | 'yellow fever'/exp/mj OR 'yellow fever' OR 'yellow fever':ti OR 'yellow-fever':ti |
| 2 | 'aedes'/exp/mj OR 'aedes':ti OR 'haemagogus':ti OR 'sabethes':ti |
| 3 | 'mosquito vector'/exp/mj OR (('mosquito'/exp/mj OR 'culicidae':ti OR 'mosquito':ti OR 'mosquitoes':ti) AND 'disease*':ti) OR 'mosquito borne disease*':ti OR 'mosquito-borne disease*':ti OR 'vector borne disease*':ti OR 'vector-borne disease*':ti |
| 4 | #1 OR #2 OR #3 |
| 5 | ((future NEAR/3 distribution):ti) OR ((future NEAR/3 spread):ti) OR 'projected distribution*':ti |
| 6 | #4 AND #5 |
| 7 | 'future':ti OR 'predict*':ti OR 'forecast*':ti OR 'evolution':ti OR 'evolv*':ti OR 'expansion':ti OR 'expand*':ti OR 'model*':ti OR 'simulat*':ti OR 'project*':ti OR 'redistribut*':ti OR 're-emerg*':ti OR 'chang*':ti |
| 8 | #4 AND #7 |
| 9 | 'meteorological phenomena'/exp/mj OR 'meteorological factor*':ti OR 'climate'/exp/mj OR 'climate change'/exp/mj OR 'climate change*':ti OR ((climate NEAR/2 alteration):ti) OR ((climate NEAR/2 alterations):ti) OR 'climate projection*':ti OR climate:ti OR 'climatic':ti OR ((climate NEAR/2 variability):ti) OR ((climate NEAR/2 variation):ti) OR 'climate crisis':ti OR 'climate emergency':ti OR 'climatic factor*':ti OR 'climatic driver*':ti OR 'anthropogenic climate change':ti OR 'weather'/exp/mj OR ((weather NEAR/2 alteration):ti) OR ((weather NEAR/2 alterations):ti) OR ((weather NEAR/2 pattern):ti) OR ((weather NEAR/2 patterns):ti) OR 'extreme weather'/exp/mj OR 'extreme weather':ti OR 'meteorological concept':ti OR 'air patterns':ti OR 'el nino'/exp/mj OR 'enso':ti OR 'el nino':ti OR 'la nina':ti OR 'temperature'/exp/mj OR temperature*:ti OR 'humidity'/exp/mj OR 'humidity':ti OR 'precipitation':ti OR 'rain'/exp/mj OR rain*:ti OR 'drought'/exp/mj OR drought*:ti OR 'flooding'/exp/mj OR flood*:ti OR 'global warming potential'/exp/mj OR 'global warming':ti OR 'greenhouse effect'/exp/mj OR 'greenhouse effect':ti OR 'carbon emissions':ti |
| 10 | 'environmental protection'/exp/mj OR ((exploitation NEAR/1 natural NEAR/1 resources):ti) OR 'deforest*':ti OR ((forest NEAR/2 conversion):ti) OR 'desertification':ti OR ((land NEAR/2 use NEAR/2 change):ti) OR 'land use':ti OR 'agriculture'/exp/mj OR 'farming':ti OR 'agricultural development':ti OR ((land NEAR/2 cover):ti) OR 'environmental degradation':ti OR ((environmental NEAR/2 degradation):ti) OR ((forest NEAR/2 fragmentation):ti) OR 'zoonosis'/exp/mj OR spillover:ti OR 'urbanization'/exp/mj OR 'urbani*ation':ti OR 'urban expansion':ti OR 'socioeconomics'/exp/mj OR 'health disparity'/exp/mj OR 'socioeconomic':ti OR 'population projection'/exp/mj OR 'population projection*':ti OR 'population growth estimat*':ti OR 'population growth'/exp/mj OR 'population growth':ti OR 'population dynamics'/exp/mj OR 'rural urban migration*':ti OR 'migration'/exp/mj OR ((human NEAR/1 movement):ti) OR 'movement of humans':ti OR 'human mobility':ti OR 'human migration':ti OR 'migration of humans':ti OR 'health transition':ti OR 'travel'/exp/mj OR 'travel':ti OR 'trade':ti OR 'trade route*':ti OR 'behavior'/exp/mj OR 'human behavio*r*':ti OR 'human activit*':ti OR 'mosquito control'/exp/mj OR 'mosquito* control*':ti OR ((mosquito NEAR/2 control):ti) OR ((mosquitoes NEAR/2 control):ti) OR 'vector* control*':ti OR (vector NEAR/2 control) OR ((vectors NEAR/2 control):ti) OR 'immunization'/exp/mj OR 'immuni*ation':ti OR 'vaccination'/exp/mj OR 'vaccin*':ti OR 'prevent*':ti |
| 11 | #8 AND #9 |
| 12 | #8 AND #10 |
| 13 | #6 OR #11 OR #12 |
| 14 | #13 AND [embase]/lim |

## Cochrane CENTRAL

| **ID** | **Search** |
| --- | --- |
| #1 | MeSH descriptor: [Yellow Fever] explode all trees |
| #2 | yellow fever:ti |
| #3 | MeSH descriptor: [Mosquito Vectors] explode all trees |
| #4 | MeSH descriptor: [Culicidae] explode all trees |
| #5 | ((mosquito NEXT borne NEXT disease*) OR (vector NEXT borne NEXT disease*)):ti |
| #6 | (#4 AND disease*:ti) |
| #7 | #1 OR #2 OR #3 OR #5 OR #6 |
| #8 | ((future NEAR/3 distribution) OR (future NEAR/3 spread) OR (projected NEXT distribution*)):ti |
| #9 | #7 AND #8 |
| #10 | (future OR predict* OR forecast* OR evolution OR evolv* OR expansion OR expand* OR model* OR simulat* OR project* OR redistribut* OR re-emerg* OR chang*):ti |
| #11 | #7 AND #10 |
| #12 | MeSH descriptor: [Air Movements] explode all trees |
| #13 | MeSH descriptor: [Climate] explode all trees |
| #14 | MeSH descriptor: [Climate Change] explode all trees |
| #15 | MeSH descriptor: [Climatic Processes] explode all trees |
| #16 | MeSH descriptor: [Droughts] explode all trees |
| #17 | MeSH descriptor: [El Nino-Southern Oscillation] explode all trees |
| #18 | MeSH descriptor: [Extreme Weather] explode all trees |
| #19 | MeSH descriptor: [Floods] explode all trees |
| #20 | MeSH descriptor: [Global Warming] explode all trees |
| #21 | MeSH descriptor: [Greenhouse Effect] explode all trees |
| #22 | MeSH descriptor: [Humidity] explode all trees |
| #23 | MeSH descriptor: [Meteorological Concepts] explode all trees |
| #24 | MeSH descriptor: [Rain] explode all trees |
| #25 | MeSH descriptor: [Temperature] explode all trees |
| #26 | MeSH descriptor: [Weather] explode all trees |
| #27 | ("anthropogenic climate change" OR "carbon emissions" OR (climate NEAR/2 alteration) OR (climate NEAR/2 alterations) OR (climate NEXT change*) OR "climate crisis" OR "climate emergency" OR (climate NEXT projection*) OR (climate NEAR/2 variability) OR (climate NEAR/2 variation) OR climate OR (climatic NEXT driver*) OR (climatic NEXT factor*) OR climatic OR drought* OR "el nino" OR "ENSO" OR "extreme weather" OR flood* OR "global warming" OR "greenhouse effect" OR humidity OR "la nina" OR (meteorological NEXT factor*) OR precipitation OR rain* OR temperature* OR (weather NEAR/2 alteration) OR (weather NEAR/2 alterations) OR (weather NEAR/2 pattern) OR (weather NEAR/2 patterns)):ti |
| #28 | #12 OR #13 OR #14 OR #15 OR #16 OR #17 OR #18 OR #19 OR #20 OR #21 OR #22 OR #23 OR #24 OR #25 OR #26 OR #27 |
| #29 | MeSH descriptor: [Agriculture] explode all trees |
| #30 | MeSH descriptor: [Behavior] explode all trees |
| #31 | MeSH descriptor: [Conservation of Natural Resources] explode all trees |
| #32 | MeSH descriptor: [Health Transition] explode all trees |
| #33 | MeSH descriptor: [Human Migration] explode all trees |
| #34 | MeSH descriptor: [Immunity] explode all trees |
| #35 | MeSH descriptor: [Mosquito Control] explode all trees |
| #36 | MeSH descriptor: [Population Dynamics] explode all trees |
| #37 | MeSH descriptor: [Population Forecast] explode all trees |
| #38 | MeSH descriptor: [Population Growth] explode all trees |
| #39 | MeSH descriptor: [Socioeconomic Disparities in Health] explode all trees |
| #40 | MeSH descriptor: [Socioeconomic Factors] explode all trees |
| #41 | MeSH descriptor: [Travel] explode all trees |
| #42 | MeSH descriptor: [Urbanization] explode all trees |
| #43 | MeSH descriptor: [Vaccines] explode all trees |
| #44 | MeSH descriptor: [Zoonoses] explode all trees |
| #45 | ("agricultural development" OR deforest* OR desertification OR (environment NEAR/2 degradation) OR "environmental degradation" OR (exploitation NEAR/1 natural NEAR/1 resources) OR farming OR (forest NEAR/2 conversion) OR (forest NEAR/2 fragmentation) OR (human NEXT activit*) OR (human NEXT behavio*r*) OR "human migration" OR "human mobility" OR (human NEAR/1 movement) OR immuni*ation OR (land NEAR/2 cover) OR (land NEAR/2 use NEAR/2 change) OR "land use" OR (migration NEAR/1 humans) OR (mosquito NEAR/2 control) OR (mosquito* NEXT control*) OR (mosquitos NEAR/2 control) OR (movement NEAR/1 humans) OR (population NEXT growth NEXT estimat*) OR "population growth" OR (population NEXT projection*) OR prevent* OR (Rural NEXT Urban NEXT Migration*) OR socioeconomic OR spillover OR (trade NEXT route*) OR trade OR travel OR "urban expansion" OR urbani*ation OR vaccin* OR (vector* NEXT control*) OR (vector NEAR/2 control) OR (vectors NEAR/2 control)):ti |
| #46 | #29 OR #30 OR #31 OR #32 OR #33 OR #34 OR #35 OR #36 OR #37 OR #38 OR #39 OR #40 OR #41 OR #42 OR #43 OR #44 OR #45 |
| #47 | #11 AND #28 |
| #48 | #11 AND #46 |
| #49 | #9 OR #47 OR #48 |
| **#50** | **#49 in Trials** |

# Protocol S2 Data extraction form

|  |  | **Studies** | | | | **Focus** | | | | | | | | **Population** | | | | | **Outcome 1** | | | | **Outcome 2** | | | **Outcome 3** | | | | | **Outcome 4** | | **Overall author conclusions** | | |
| --- | --- | --- | --- | --- | --- | --- | --- | --- | --- | --- | --- | --- | --- | --- | --- | --- | --- | --- | --- | --- | --- | --- | --- | --- | --- | --- | --- | --- | --- | --- | --- | --- | --- | --- | --- |
| Data extraction element |  | **Covidence ID  (ID# // First author)** | **Publication Year** | **Study type** | **Publication type** | **Focus (e.g. YFV, YF disease, vector evolution)** | **Infection/virus  assessed  (e.g. Dengue.YF)** | **Vector assessed** | **Study methodology** | **Model type** | **Analysis in relation to (e.g. climatic factors, human behaviour)** | **Covariates**  **related to**  **climatic factors** | **Covariates  related to  human behaviour** | **Geography** | **Setting** | **Endemic /  Non-endemic** | **Period of time covered (observed or projected)** | **Outcome assessed** | | **Stated limitations** | **Study conclusions** | **Outcome assessed** | | **Stated limitations** | **Study conclusions** | | **Outcome assessed** | **Stated limitations** | **Outcome conclusions** | **Outcomes assessed** | | **Study limitations** | | **Study conclusions** | **Study conclusions** |
|  |  |  |  |  |  |  |  |  |  |  |  |  |  |  |  |  |  |  |  |  |  |  |  |  |  |  |  |  |  |  |  |  |  |  |  |
| Instructions to reviewers |  | *This information will be automatically added by Covidence* |  | *Choose study type* | *Choose publication type* |  | *Choose infection/virus assessed* |  |  |  | *Tick either or both* | *Tick as many as apply* | *Tick as many as apply* | *State countries covered  in the study* | *State setting by the study/analysis/model* | *Tick either or both* | *State period of time covered by the study/analysis/model* | *State the first/main outcome assessed in the analysis* | | *Copy/paste text as  reported by authors* | *Copy/paste text as  reported by authors* | *State the second outcome assessed in the analysis* | | *Copy/paste text as  reported by authors* | *Copy/paste text as  reported by authors* | | *State the third outcome assessed in the analysis* | *Copy/paste text as  reported by authors* | *Copy/paste text as  reported by authors* | *State the fourth outcome assessed in the analysis* | | *Copy/paste text as  reported by authors* | | *Copy/paste text as  reported by authors* | *Copy/paste text as  reported by authors* |
| Format  of data extraction element |  |  |  | *Choose study type from list of options:  • Predictive modelling study • Case study • Interventional study*  *• Observational study* | *Choose publication type from list of options:  • Original research article • Congress abstract* | *Free text* | *Choose as many options as apply from list: • Dengue • YF • Chikungunya • Zika • Rift Valley Fever (RFV) • Mayaro Virus (MAYV)  • Ross River Virus* | *Free text* |  |  | *• Climatic factors*  *• Human behaviour* | *Choose as many options as apply from list: • Temperature • Mean temperature • Min temperature • Max temperature • Humidity  • Precipitation/rainfall • Total rainfall • Mean rainfall • Max rainfall • Greenhouse gas emissions/ CO_2_ concentration • Wind  • El Niño Southern Oscillation Index • Soil moisture (water stress/wetness)* | *Choose as many options as apply from list:* ***Demographic factors*** *• Population density • Age • Air travel • Altered travel and trade routes • Human daily mobility • Vaccination coverage*  ***Socio-economic factors*** *• Gross domestic product • Household income*  ***Ecology*** *• Location of breeding sites • Vector control and prevention*  ***Environmental factors*** *• Vegetation • Elevation/altitude • Urbanization • Distance to roads, road density • Land use/land cover • Deforestation and forest fragmentation*  ***Spatiotemporal incidence*** *• Case count across time periods and neighbouring regions* | *Free text* | *Multiple choice list: • Global • Regional • National • Sub-national* | *• Endemic • Non-Endemic* | *Free text* | *Free text* | | *Free text* | *Free text* | *Free text* | | *Free text* | *Free text* | | *Free text* | *Free text* | *Free text* | *Free text* | | *Free text* | | *Free text* | *Free text* |

# Protocol S3 GenAI usage

GenAI use for data analysis and presentation, and in the manuscript.

The data were analysed according to category, i.e. “vector distribution and abundance” (Table S2 + Table S3), “other vector parameters” (Table S4 + Table S5), “yellow fever infection and disease transmission” (Table S6 + Table S7 + Table S8). A total of five separate analyses were run in order to refine the prompts used. The final set of prompts used were as follows:

- Vector distribution and abundance
  - Using the information in these two tables, can you summarize the main consequences for the distribution and abundance of yellow fever vectors that are directly caused by (1) climate change or (2) human behaviour? Do not include covariates (e.g. things such as temperature which vary with climate change). Please also do not consider other vector parameters (e.g. evolution) or yellow fever infection or transmission. Please note how many of the 29 studies are in agreement on each point, and report how much variability there is amongst the studies for each of the consequences you list.
  - Can you now summarize the main covariates (e.g. things such as temperature which vary with climate change, or land use which varies with human behaviour) for vector distribution and abundance that these papers suggest? Do not include consequences (i.e. things caused directly by climate change). Please note how many of the 29 studies are in agreement on each point, and report how much variability there is amongst the studies for each of the consequences you list.
- Other vector parameters
  - Using the information in these two tables, can you summarize the main consequences for vectors (do not include vector distribution or vector abundance) that are directly caused by (1) climate change or (2) human behaviour? Do not include covariates (e.g. things such as temperature which vary with climate change). Please note how many of the 14 studies are in agreement on each point, and report how much variability there is amongst the studies for each of the consequences you list.
  - Can you now summarize the main covariates (e.g. things such as temperature which vary with climate change) for vector parameters other than distribution and abundance that these papers suggest? Do not include consequences (i.e. things caused by climate change). Please note how many of the 14 studies are in agreement on each point, and report how much variability there is amongst the studies for each of the consequences you list.
- Yellow fever infection and disease transmission
  - Using the information in these 3 tables, can you summarize the main consequences for yellow fever infections and disease transmission that are directly caused by (1) climate change or (2) human behaviour? Do not include covariates (e.g. things such as temperature which vary with climate change). Please note how many of the seven studies are in agreement on each point, and report how much variability there is amongst the studies for each of the consequences you list.
  - Can you now summarize the main covariates (e.g. things such as temperature which vary with climate change) for yellow fever infections and disease transmission that these papers suggest? Do not include consequences (i.e. things caused by climate change). Please note how many of the seven studies are in agreement on each point, and report how much variability there is amongst the studies for each of the consequences you list

GenAI was used only to summarise content from completed extraction tables and to generate draft descriptive summaries. All final trend categories, study allocations, and manuscript interpretations were determined manually by the review team.

All studies listed as supporting each final trend were manually verified against the extraction tables before inclusion in the manuscript. A manual comparison exercise was performed to confirm that AI-assisted summaries did not materially alter the direction of the pre-existing manual synthesis. GenAI was used occasionally during drafting of the manuscript, particularly for preparation of the abstract. The outputs were always subsequently edited by a medical writer and checked for accuracy.

# Table S1 Characteristics of the included modelling studies

| **Reference** | **Model approach** | **Model type** | **Input data** |
| --- | --- | --- | --- |
| Aliaga-Samanez 2024 | Global climate projection of future *Aedes aegypti* and *Aedes albopictus* spatial distribution using pre-existing favorability models by substituting future climate variables while holding non-climatic variables constant | Correlative species distribution model based on favorability function (logistic regression-derived) | Baseline favorability models (2001-2017), future climate data from 5 GCMs under RCP 4.5/8.5 scenarios via CHELSA, and static non-climatic variables (human concentration, infrastructure, livestock, topography, agriculture, ecosystem types) |
| Bonnin 2022 | Process-based compartmental modeling using ordinary differential equations to simulate current and future *Aedes* aegypti and *Aedes albopictus* population dynamics across 10 life cycle stages in Southeast Asia | Mechanistic population dynamics model. | Daily temperature and precipitation from 9 CMIP6 climate models under 4 SSP scenarios, species occurrence data, and land cover/topography data for species distribution weighting |
| Campbell 2015 | Global potential distributions and future distributional potential of *Aedes aegypti* and *Aedes albopictus* in relation to climatic variation worldwide | Class Correlative ecological niche models calibrated in MAXENT and transferred to future-climate scenarios | Primary occurrence data for *Aedes aegypti* and *Aedes albopictus*, WorldClim bioclimatic variables, CMIP4 future-climate projections, and an *Aedes* sampling-bias layer |
| Capinha 2014 | Global macroclimatic envelope and future distribution of *Aedes aegypti* | Correlative climate-envelope model using alpha-shapes | Global occurrence records, temperature and precipitation variables, and future climate projections (A2a/B2a scenarios) |
| Donelly 2016 | Past, present, and future habitat suitability for *Aedes aegypti* and *Aedes albopictus* in the continental United States | Process-based, stage-structured matrix population model | Daily average temperatures for the United States on a 4-km grid and CMIP5 future climate projections |
| Escobar 2016 | Present-day and future potential distributions of 14 disease vector species in Ecuador | Ecological niche modelling with MAXENT and future climate projections | Global vector occurrence records, remote sensing environmental variables, climate data, and future CliMond climate projections (A2 scenario) |
| Kamal 2018 | Global potential distributions and future distributional changes of *Aedes aegypti* and *Aedes albopictus* | Ecological niche model (MAXENT) | Occurrence records, WorldClim bioclimatic variables, and future climate projections from 9 GCMs under four RCP scenarios |
| Khormi 2014 | Potential global distribution of *Aedes aegypti* under current and future climate conditions | CLIMEX eco-climatic niche model using the EI | Species distribution records, temperature, precipitation, humidity, and future climate projections from CSIRO-Mk3.0 and MIROC-H under A1B/A2 scenarios |
| Laporta 2023 | Fundamental niche modeling to estimate current and future global distributions of *Aedes aegypti* and *Aedes albopictus* under a climate change scenario of regional rivalry | Correlative species distribution model using BRTs | Global occurrence records from VectorMap, monthly temperature and precipitation variables from WorldClim v2.1, and future climate projections from the GFDL-ESM4.1 model under the SSP3-7.0 scenario for 2021–2040, 2041–2060, 2061–2080, and 2081–2100 |
| Lippi 2019 | Ecological niche modeling to estimate current and future habitat suitability of *Aedes aegypti* larvae and assess potential geographic shifts in exposure risk under climate change in Ecuador | Correlative ecological niche model using the Genetic Algorithm for Rule-Set Production (GARP) | Larval surveillance records, elevation, human population density, bioclimatic variables, and future climate projections from BCC-CSM-1, CCSM4, and HADGEM2-ES under RCP 2.6, 4.5, 6.0, and 8.5 scenarios projected to 2050 |
| Liu 2019  (China) | Ecological niche modeling to estimate current and future distributions of *Aedes aegypti* and *Aedes albopictus* and evaluate changes in exposed area and population under climate change in Mainland China | Correlative species distribution model (MaxEnt) | Species occurrence records, bioclimatic variables, land-use variables, population density data, and future climate projections from the BCC-CSM1-1 model under RCP 2.6, 4.5, and 8.5 scenarios for the 2030s and 2050s |
| Liu 2019  (Taiwan) | Ecological niche modeling to predict current and future habitat suitability and potential range expansion of *Aedes aegypti* in Taiwan under climate and land-use change scenarios | Correlative species distribution model (MaxEnt) | *Ae. aegypti* occurrence records, bioclimatic variables, land-use variables, and future climate projections from the BCC-CSM1-1 model under RCP 2.6, 6.0, and 8.5 scenarios for the 2030s, 2050s, and 2070s |
| Liu-Helmersson 2019 (Global) | Modelling of current and potential future global spatial distribution of *Aedes aegypti* | Process-based mathematical modelling of the lifecycle of *Aedes aegypti* and potential population density based on climate, economic and human population factors | Mosquito morphological and lifecycle data from the literature, climate data from the Climate Research Unit online database and ISIMIP, RCP and SSP scenarios, socioeconomic data including yearly global GDP and human population density from ISIMIP |
| Liu-Helmersson 2019 (Europe) | Modelling of future population growth rate of *Aedes aegypti* to predict future spatial distribution within Europe | Process-based mathematical modelling of the lifecycle of *Aedes aegypti* and vector invasion risk based on environmental conditions as modelled by five GCMs | Mosquito morphological and lifecycle data from the literature, climate data from the Climate Research Unit online database, RCP scenarios, annual global human population data from ISIMIP |
| Outammassine 2022 | Ecological niche modelling of present and future global spatial distributions of *Aedes japonicus, Aedes vexans* and *Aedes vittatus* | MaxEnt modelling of mosquito species spatial distribution based on current climate conditions and predicted future conditions as modelled by diverse GCMs | Global occurrence data for *Aedes japonicus, Aedes vexans,* and *Aedes vittatus* from the Global Biodiversity Information Facility and the literature, climate data from WorldClim, RCP scenarios and GCMs |
| Portilla Cabera 2020 | Modelling of present and future spatial distribution of *Aedes aegypti* in Colombia | MaxEnt modelling of mosquito spatial distribution in current climate conditions and predicted future conditions as modelled by SDMtoolbox 2.0 | Country-level occurrence data for *Aedes aegypti* from the Global Biodiversity Information Facility, climate data from WorldClim, RCP scenarios, altimetry data from the Shuttle Radar Topography Mission |
| Romero-Alvarez 2017 | Ecological niche modelling of current and future spatial distributions of vectors associated with various infectious diseases in Ecuador | Ecological niche modelling using a logistic-regression like algorithm with species-specific  configurations selected via an information theory metrics | Worldwide occurrence reports of 14 infectious disease vector species and climate information |
| Sedaghat 2023 | Modelling of future habitat suitability for *Aedes albopictus* and *Aedes aegypti* in Iran | MaxEnt modelling of spatial distribution of mosquito species based on historic occurrence data and predicted future climate characteristics | Historical mosquito occurrence data from the literature, climate data from WorldClim, RCP scenarios |
| Trájer 2021 | Modelling of historic, current and predicted future distribution of *Aedes aegypti* in the Mediterranean, based on climate factors and mosquito biology | Boolean algebra-based modelling of environmental requirements-based  distribution areas of *Aedes aegypti*, utilising temperature- and precipitation-related bioclimate variables, and mosquito development time and survival rate data | Georeferenced bioclimate data from WorldClim, RCP scenarios, previously published data on mosquito occurrence and morphology |
| Williams 2014 | Modelling of the effect of different climate change scenarios on *Aedes aegypti* morphology and distribution in north Queensland, Australia | Simulation of mosquito population dynamics using the CIMSiM software program, incorporating predicted daily data from two climate emission scenarios | CIMSiM-sourced entomological data for annual variation in Dengue virus transmission in Australia, field data and previously published data on water flux and food delivery to mosquito habitats, downscaled data based on carbon emission model outputs from the literature |
| Carbajo 2019 | Species distribution modelling of *Aedes aegypti* in Argentina in relation to environmental and demographic factors, and projected future distribution based on differing future climate and human demographic scenarios | Generalised linear mixed modelling and quasibinomial proportional general linear modelling of historical and future mosquito distributions | Historical mosquito occurrence data from the authors, the literature and local health authorities, climate data from Climatic Research Unit Time-Series Version 3.24.01, population data from national demographic censuses, precipitation and altitude data from the Instituto Geografico Nacional |
| Equihua 2017 | Ecological niche modelling of current and future distribution of *Aedes aegypti* in mountainous regions in Mexico, based on climate data and historical mosquito occurrence | Fitted ecological niche modelling of *Aedes aegypti* based on mosquito occurrence data from the literature, health agencies and the present study and climate variables | Entomological sample data from the present study, mosquito occurrence data from the literature and health agencies, published historical climate data and RCP scenarios |
| Gorris 2024 | Ecological niche modelling and projection of the future geographic ranges of *Aedes aegypti* and *Aedes albopictus*, as well as several *Culex* species, across the Americas | Machine learning, MaxEnt-based modelling of future ecological niches and geographic distributions of each assessed mosquito species, based on land use projections from five GCMs and climate projections based on a delta-climate change approach | Mosquito presence data (2000–2019) from VectorBase and VectorMap, annual temperature and precipitation data from the TerraClimate dataset, land usage data from the GCAM-Demeter land use dataset, RCP and SSP scenarios |
| Kearney 2009 | Modelling of spread of *Aedes aegypti* in Australia due to predicted effects of future climate changes on breeding site availability | Mixed modelling of egg survivorship in dry periods via logistic regression, availability and temperature of water sources via coupled transient-state energy and mass balance equations, evolutionary potential of egg desiccation using standard quantitative genetic models, a 9′′ Digital Elevation Model to estimate elevation and temperature in Australia, and Ozclim v2 to predict changes in monthly climate factors | Mosquito physiological data from previous publications, breeding container property data from a previous publication, climate data from previous publications |
| Khan 2020 | Modelling of spread of *Aedes aegypti* and *Aedes albopictus* in Canada and the USA under current and predicted future climates | BRT-based modelling of future climate factors and urban development and to predict future mosquito distribution | Mosquito occurrence data from multiple databases and governmental institutions, temperature and vegetation index data from the Moderate Resolution Imaging Spectroradiometer Platform, average precipitation data from the Modern-Era Retrospective  Analysis for Research and Applications, urban land usage data from the Global  Rural-Urban Mapping project, RCP and SSP scenarios and projected global population grids for 2011 – 2100 from a previous publication |
| Kraemer 2019 | Modelling of the future distribution of suitable environments for *Aedes aegypti* and *Aedes albopictus* and simulating the future spread of both species in the United States and Europe based on past dispersal patterns and human movement data | Mixed model estimation using BRT models to fit mosquito occurrence data and predict future distributions and GCMs to predict climate factors | Global mosquito occurrence data collated from various published and unpublished sources for an earlier study, RCP scenarios, time-series occurrence records for *Aedes aegypti* and *Aedes albopictus* from previous studies, workforce commuting flow data in the USA, mobile phone or CDR data from France, Portugal and Spain, human movement data from a large location-based service in China, GPS data for all taxis in Changsha City, China, and mobile phone data from Namibia |
| Santos 2022 | Modelling of current and predicted future distribution of climates suitable for *Aedes aegypti* on Madeira Island in conjunction with land use and estimated future human settlement | Mixed model estimation of climate suitability using BRT, GAM, GLM and RFT. A second set of mixed models (not including a GAM) was used to predict land use and human settlement patterns | Species distribution data from Madeira’s Regional Directorate for Health and the Global Biodiversity Information Facility, global scale climate data from the CHELSA database, RCP scenarios, land use data from the Regional Administration of  Environment and Climate Changes of the Madeira government and data on population density and residential housing from Statistics Portugal |
| Wint 2022 | Spatial distribution modelling of current and predicted future distribution of *Aedes aegypti* in Europe and neighbouring areas | Spatial distribution modelling using RFTs and BRTs to model presence of *Aedes aegypti* based on climate factors | Historical (<1980) and contemporary (≥1980) data for distribution of *Aedes aegypti,* historic and projected future climate data, historic human population data and SSP scenarios |
| Iwamura 2020 | Modelling of global current and predicted future *Aedes aegypti* life cycle stage viability and life cycle completions based on climatic factors | Spatially explicit phenology model incorporating the life cycle stages of *Aedes aegypti* based on a pre-existing phenology modelling framework, linked to gridded climate data and using RCP scenarios to project future trends | *Aedes aegypti* growth and biological data derived from the literature, gridded climate data, RCP scenarios |
| Piovezan-Borges 2020 | Modelling of adult emergence pattern of *Aedes* aegypti in the Western Amazon region of Brazil according to simulated climate change scenarios | Linear mixed modelling of *Aedes aegypti* adult emergence pattern with replicates as a random factor and simulated climate change scenarios and predation risk as fixed factors | Experimental data from *Aedes aegypti* larva subjected to differing mean temperatures, CO_2_ concentrations and levels of predation |
| Rose 2020 | Ecological modelling of current and future mosquito behaviour and host selection across various climate and human population density change predictions in Africa | Multi-stage ecological modelling of mosquito behaviour at an individual and population level via a series of individual models | Morphological and genetic data from captured mosquitos at sampling sites, RCP scenarios and population projection data |
| Davis 2021 | Suitable condition index for *Aedes aegypti* and *Aedes albopictus* to predict current and future geographic distribution in the Asia-Pacific region | Climate-based suitable condition index developed based on a previous temperature- dependent transmission model of the optimum temperature range for *Aedes aegypti-* and *Aedes albopictus*-mediated dengue virus transmission | Geographic boundary data for countries in Southeast Asia, gridded historical climate data for 2005-2018, projected values for temperature and average monthly precipitation, RCP scenarios, suitable temperature ranges for dengue vectorial capacity |
| Gaythorpe 2020 | Projection of current and future transmission intensity and burden of YFV in Africa according to temperature suitability for YF | Modified pre-existing GLM of YF occurrence to include a nonlinear temperature suitability metric, combined with a Bayesian hierarchical model of YF occurrence data to account for uncertainty | Data on YF outbreaks from 1984 – 2020, surveys of seroprevalence in Central and East Africa, historic vaccination occurrence data, mean annual rainfall, average temperature, temperature range, RCP scenarios, YF-specific sources of information on extrinsic incubation period, vector mortality and bite rate for *Aedes aegypti* |
| Ryan 2019 | Mechanistic model of months of suitability and global populations at risk of infection with *Aedes aegypti-* and *Aedes albopictus*- borne infections in different climate change scenarios | Temperature-dependent model of R_0_ linked to current climate data and projected future climates from four GCMs and RCPs, as well as gridded population count data | Mosquito, viral and human data linked to R_0_, current climate data (mean monthly temperature), GCMs, RCPs, climate model output data for future scenarios and population count data |
| Sadeghieh 2021 | Modelling of YFV transmission in humans and projection of future YF outbreaks in Brazil according to differing future regional climate scenarios | Modification of a pre-existing mathematical compartment model describing chikungunya virus dynamics based on data for a variety of parameters from the literature on the 2017/18 YF virus outbreak in Brazil | Temperature data from RCMs, RCP scenarios, mosquito extrinsic incubation period, death rate and development rate, transmission rate (human to mosquito and vice versa), proportion of symptomatic humans, human death rate, human incubation period, duration of infectiousness |
| Monaghan 2018 | Projection of future global occurrence patterns of *Aedes aegypti* and estimated number of humans exposed to infection in the late 21^st^ century based on two distinct climate change outcomes and two population pathways | Combination of four occurrence patterns for *Aedes aegypti* and climatology data from 1950-2000 to create predicted maps of *Aedes aegypti* occurrence patterns in the late 21^st^ century using future climate projections from a fully coupled atmosphere-ocean global climate model, forced in accordance with two RCPs. The impact of future population changes on human exposure was modelled using gridded global population projections from two SSPs | SSP and RCP scenarios, occurrence patterns of *Aedes* aegypti and global climatology data from 1950-2000 |
| Rohat 2020 | Projection of future population exposure to *Aedes*-borne virus transmission in the conterminous US based on combinations of emissions and socioeconomic/demographic scenarios with projections of Aedes-borne virus transmission risk | Combination of SSP-RCP scenarios with a pre-existing temperature-driven empirically parameterised model of viral transmission and four GCMs of baseline and future temperature projections to estimate future cumulative monthly transmission risk | SSP and RCP scenarios, projections of *Aedes*-borne virus transmission risk at baseline and for several future time periods, population projections for particular groups considered to be at special risk of infection with Aedes-borne diseases (total population, individuals aged ≥65 years, individuals aged 5–14 years, urban population, individuals who have occupations in which > 70% of the work performed is outside, population in poverty) |

ANOVA, analysis of variance; BRT, boosted regression tree; CDR, call detail record; CHELSA, Climatologies at High resolution for the Earth's Land Surface Areas; CIMSiM, Container-inhabiting Mosquito Simulation; EI, ecoclimatic index; GAM, generalised additive model; GCM, general circulation model; GDP, gross domestic product; GLM, generalised linear model; GPS, Global Positioning System; ISIMIP, Inter-Sectoral Impact Model Intercomparison Project; MaxEnt, maximum entropy; RCM, regional climate model; RCP, Representative Concentration Pathway; RFT, random forest tree; SSP, Shared Socioeconomic Pathway; YF, yellow fever; YFV, yellow fever virus

# Figure S1 Characteristics of the included studies

Included studies were those containing data relating to predicting / modelling / forecasting trends over time of YFV and YF disease (e.g., burden of disease, number of human / non-human cases, disease severity and mortality, and geographical distribution), YF vector (*Aedes, Haemogogus* or *Sabethes* mosquitoes) evolution, changes in mosquito development / distribution and changes in YF transmission in relation to climatic factors, and / or in relation to human behaviour. Searches were conducted from database inception up to 11 September 2024 in the following databases: (1) MEDLINE via PubMed, (2) Embase via Embase.com, (3) Cochrane CENTRAL via the Cochrane Library. Manual searches were conducted on 13 September 2024 of congress abstract books (last 2 years of Conference of the International Society of Travel Medicine (CISTM), American Society of Tropical Medicine & Hygiene Congress (ASTMH), Pan-American Dengue Research Network (PanDengue Net) Meeting) to identify additional studies. The Journal of Climate Change and Health was also searched manually for additional relevant articles.
YF, yellow fever, YFV, yellow fever virus.

# Figure S2 Prevalence of yellow fever vector species in the literature dataset

Only the twelve species recognised as YFV vectors were included in the analysis; publications reporting data for *Ae. albopictus* only were excluded as it is not officially recognised as a vector of YFV. The final analysis included 42 publications in total.

*Ae*., *Aedes*; *Hg*., *Haemagogus*; *S*., *Sabethes*; YFV, yellow fever virus*.*

# Table S2 Studies on the effects of climatic factors on vector distribution and abundance

| **Reference** | **Study** | **Vector** | **Geography** | **Covariates** | **Key finding** |
| --- | --- | --- | --- | --- | --- |
| Aliaga-Samanez 2024 | PMS | Various^a^ | Global | CO_2_ emission | Favourable areas for the presence of dengue and yellow fever vectors show little differences in the future compared with the baseline models, with changes being perceptible only at regional scales. Global favourability values could decrease slightly in the near future but might increase in the distant future. This increase is expected to affect principally the Amazon Basin and West and Central Africa. In some locations of Europe, the models suggest a reestablishment of *Ae. aegypti*. |
| Bonnin 2022* | PMS | *Ae. aegypti;  Ae. albopictus* | South-East Asia  *Non-endemic* | Air temperature, precipitation | Seasonal dynamics of vector densities are mainly driven by precipitation, presenting with symmetrical model outputs from field surveys in Phnom Penh and Vientiane. On a continental scale in Southeast Asia, during summer months, *Ae. aegypti* showed elevated densities in lowlands and coastal areas, driven by its association to urban habitat. In wintertime, *Ae. aegypti* densities were much lower over the domain due to lower temperature and precipitation. These results contrast with previous results, which brings new insight on the future impacts of climate change on *Aedes* densities. |
| Campbell 2015 | PMS | *Ae. aegypti;  Ae. albopictus* | Global | Temperature and precipitation | Contemporary model predictions reflected well-known global distributions of each of the two species, although there was a markedly broader distributional potential for *Ae. aegypti* than for *Ae. albopictus* in Australia and other tropical and subtropical regions. The authors’ future-climate projections indicate considerable potential for shifting establishment as follows: (i) more broadly in eastern North America, (ii) farther south in southern South America (particularly in *Ae. aegypti*) (iii) locally northward into southern Europe, (iv) more broadly in Central Africa, (v) more broadly in East. These results indicate complex global rearrangements of potential distributional areas, which are likely to translate into actual distributional shifts. |
| Capinha 2014 | PMS | *Ae. aegypti* | Global | Temperatures, water availability, fossil fuel availability and use | Climatic conditions found at latitudes higher than ~45°N or ~35°S remained almost unanimously unoccupied by the species. Below these extremes, the species encompassed mainly climatic combinations typical of tropical areas, but also some warm temperate climates such as those found in Southwest and Southeast USA, Uruguay, Northeast Argentina, the Mediterranean basin, Northern India, East China, and East Australia. The majority of hot arid climates remained unoccupied, as well as the climates found in areas at higher altitudes. Over the next decades, wide areas beyond the species’ maximum range will present potentially suitable macroclimates, including those found in Europe, the USA, Southern Africa, and Central Australia, but will depend on the vectors being introduced and the availability of favourable domestic environments. |
| Donnelly 2016 | PMS | *Ae. aegypti;  Ae. albopictus* | USA  *Non-endemic* | Temperature | Much of the southern USA is suitable for *Ae. aegypti* year-round; however, a surprisingly large proportion of the USA in addition to the southern states is suitable for positive population growth for much of the year. While the amount of suitable habitat in some regions of the USA has contracted within the past 50 years, the range of suitable habitat for *Ae. aegypti* has expanded in many regions across the country. Additionally, IPCC CMIP5 model projections of future climate change suggest that climate change will reshape the range of *Ae. aegypti* in the USA, and potentially the risk of the viruses they transmit. Understanding the range of these mosquitoes should be considered a high priority for public health officials and vector control agencies. |
| Escobar 2016 | PMS | Various^a^ | Ecuador  *Endemic* | Temperature, annual precipitation | High risk for exposure to arboviruses vectors was found across the coastal region of western Ecuador and in the Amazonian lowlands in the northeast. Additional areas of exposure risk to vectors were found across valleys of the western region of the Andes mountains; the highlands exhibited reduced suitability to arbovirus vectors under present-day environmental conditions. Climate-based predictions suggest an increased risk in southern areas in the short term (i.e., 2030). Mid-term predictions suggest that by 2050 high risk will be distributed in the coast, southern lowlands and in northeastern areas neighbouring Colombia and Peru, with reduced suitability in central areas across the Andes. The general pattern revealed that human populations in the Andean highlands would be increasingly exposed to disease vectors as climate change unfold due to likely upward vector species range shifts. |
| Kamal 2018 | PMS | *Ae. aegypti;  Ae. albopictus* | Global | CO_2_ emissions, greenhouse gas emissions | The predicted potential distribution of *Ae. aegypti* and *Ae. albopictus* coincided with the current and historical known distributions of both species. Ecological niche models based on future conditions showed similarity between the overall distributional patterns of future-day and present-day conditions. *Ae.* *aegypti* was anticipated to expand to the South in East Australia in 2050 and 2070. These results have significant public health importance as a baseline for predicting the emergence of arboviral diseases transmitted by both vectors in new areas across the world. |
| Khormi 2014 | PMS | *Ae. aegypti* | Global | Temperature, precipitation and relative humidity | The present global distribution of *Aedes* is consistent with previous models, i.e., Brazil, large areas of Central America, the Caribbean islands, parts of the western and eastern coasts of Africa, the Pacific island groups, the northeast coast of Australia, western India and Bangladesh. For future climate conditions (2030 and 2070), parts of the world with strongly positive climate areas for *Ae. aegypti* are predicted to become less favourable, counterintuitively due to the warming projected for future climate scenarios. Additional etiologic factors not addressed at this level of integrated modelling must ultimately be incorporated to determine human impact on dengue fever and its vector. |
| Laporta 2023 | PMS | *Ae. aegypti;  Ae. albopictus* | Global | Temperature, precipitation | Country distribution of *Ae. aegypti* was unequal towards countries in the tropical and subtropical zones, mainly found in Brazil (n=4780; 49.1%). The relative influences of environmental variables on the fitted model for *Ae. aegypti* were 50.71% of average maximum temperature, 24.02% of average minimum temperature, and 25.27% of total precipitation. *Ae. aegypti* potential future distributions in Americas showed that the USA will not develop additional suitable areas, while most of the Amazon basin of Brazil will become unsuitable for this species in the future. |
| Lippi 2019 | PMS | *Ae. aegypti* | Ecuador  *Endemic* | Elevation, temperature, precipitation, human population density | Much of Ecuador was predicted to be suitable for the presence of *Ae. aegypti* larvae under current climatic conditions, with the notable exception of the eastern portion of the country associated with the Amazon basin and high elevation areas associated with the Andes mountain range. In 2050, slight vector range expansions into areas of higher elevation are predicted, and large portions of the eastern Amazonian basin indicating mosquito absence. Range expansions and contractions were generally consistent across climate models, with the magnitude of distribution change increasing with more extreme climate change scenarios. |
| Liu 2019 | PMS | *Ae. aegypti* | Taiwan  *Non-endemic* | Temperature, precipitation, land-use | *Ae. aegypti* is expected to establish new habitats out of its current niche range under the trend of global climate change. The extent of habitat expansion varies under different climate scenarios. Appropriate measures should be taken to prevent its expansion to a broader scale. Our study has important strategic implications for mosquito surveillance and the prevention and control of mosquito-borne diseases. |
| Liu 2019 | PMS | *Ae. aegypti;  Ae. albopictus* | China  *Non-endemic* | Temperature, precipitation, land-use | *Ae. aegypti* is predicted to expand its niche range to varying degrees under future climate scenarios. *Ae. aegypti* was modelled to expand its habitat from Guangdong, Guangxi, Yunnan and Hainan to Fujian, Jiangxi and Guizhou. Area and population exposed to mosquitoes are predicted to increase significantly. Environmental variables that have significant impact on the distribution of mosquitoes are also revealed by our model. |
| Liu-Helmersson 2019 | PMS | *Ae. aegypti* | Europe  *Non-endemic* | Temperature, precipitation, greenhouse gas emission | In a low carbon emission future (RCP2.6), we find minimal change to the current situation throughout the whole of the 21st century. In a high carbon future (RCP8.5), a large parts of southern Europe risks being invaded by *Ae. aegypti*. Our results show that successfully enforcing the Paris Agreement by limiting global warming to below 2°C significantly lowers the risk for infestation of *Ae. aegypti* and consequently of potential large-scale arboviral disease outbreaks in Europe within the 21st century. |
| Liu-Helmersson 2019 | PMS | *Ae. aegypti* | Global | Temperature, precipitation, human behaviour, greenhouse gas emissions | The change in the potential of global abundance in *A. aegypti* over the last century up to today is estimated to be an increase of 9.5% globally and a further increase of 20 or 30% by the end of this century under a low compared to a high carbon emission future, respectively. The largest increase has occurred in the last two decades, indicating a tipping point in climate-driven global abundance which will be stabilized at the earliest in the mid-twenty-first century. The realized abundance is estimated to be sensitive to socioeconomic development. |
| Marinho 2016* | OS | *Ae. aegypti* | Brazil  *Endemic* | Temperature | Our results suggest that Campina Grande, João Pessoa, and Patos will experience, respectively, maximum temperature increases of 0.030° C/year, 0.069° C/year, and 0.061° C/year, and minimum temperature increases of 0.019° C/year, −0.047° C/year, and −0.086° C/year. These projected increases will result in temperatures favourable to the *Ae. aegypti* life cycle, causing rapid population growth. Therefore, *Ae. aegypti* populations are likely to expand in the mesoregions represented by these cities. |
| Outammassine 2022 | PMS | Various^a^ | Global | Temperature, precipitation, CO_2_ emissions | By 2050, *Ae. japonicus* will have a broader potential distribution across much of Europe, the USA, western Russia and central Asia. *Ae. vexans* may be able to expand its range, especially in Libya, Egypt and southern Australia. For *Ae. vittatus,* future projections indicated areas at risk in sub-Saharan Africa and the Middle East. |
| Portilla Cabrera 2020 | PMS | *Ae. aegypti* | Colombia *Endemic* | Temperature, precipitation, land-use | For the current conditions, Colombia has ~140,612.8 square km of areas with the possible presence of the vector; however, for the future, this will be reduced by more than 30%. For the future conditions, the suitable areas for *A. aegypti* decreased compared to the present, mainly for the year 2070 under RCP scenarios 4.5 and 8.5, however, the likelihood of mosquito occurrence increases in some departments of Colombia. Areas susceptible to the presence of *A. aegypti* are affected by climate change. The Caribbean and Andean regions have a high probability of mosquito distribution; therefore, control and epidemiological surveillance are required in these areas. |
| Romero-Alvarez 2017 | PMS | *Ae. aegypti;  An. darlingi;* | Ecuador  *Endemic* | Not specified | We found a strong signal of shrink in the distribution of several vectors (e.g., *Ae. aegypti*) under future climates, and identified new areas of distribution for some species, suggesting a plausible shift to Andean regions (e.g., *An. darlingi*). From shrink and shift of vector ranges, we identified reductions of people potentially exposed to vectors, which will resemble an uncommon scenario for climate change offering a likely benefit in terms of people expose to vector-borne diseases. |
| Sedaghat 2023 | PMS | *Ae. aegypti;  Ae. albopictus* | Iran  *Non-endemic* | Temperature and precipitation | The most suitable areas for the establishment of *Ae. aegypti* are located in the southern and northern coastal areas of Iran, based on the model outputs. |
| Trájer 2021 | PMS | *Ae. aegypti* | Mediterranean  *Non-endemic* | Temperature and precipitation | The modelled present climatic suitability patterns of the mosquito show that large areas of the lower altitude Mediterranean regions, including the coastal areas of the Balkan Peninsula, South France, and large regions of the Apennines and the Iberian Peninsulas could be suitable for *Ae. aegypti.* The future (2041–2060 and 2061–2080) projections predict the potential northward shift of the northern occurrence of the species in the circum-Mediterranean and Black Sea areas. Both, the potential development time, and survival rate of *Ae. aegypti* in the late 19th and the early 20th century could be like in the present times along the Mediterranean coast. The current climatic conditions cannot explain the absence of the mosquito in wide areas of the Mediterranean and sub-Mediterranean ecoregions. The future models predict the notable increase in the development time and survival rate of the mosquito in the southern and central regions of Europe. In general, the container ports of the Alboran, Balearic, and Aegean seas seem to be the most suitable sites for the re-colonization of the mosquito, and such northern parts of the Mediterranean Sea like the Gulf of Lion, the Ligurian, and Adriatic Seas are in less extent. |
| Williams 2014* | PMS | *Ae. aegypti* | Australia  *Non-endemic* | Temperature and precipitation | Overall mosquito abundance is predicted to change, but results were equivocal for different climate change scenarios.  *Ae. aegypti* abundance is predicted to increase under the B1 emission models but decrease under the A2 emission-model scenario. |

Studies are color-coded according to whether they report (1) no expansion or regression (green), (2) low likelihood of expansion (beige), (3) high likelihood of expansion (orange). *Also assessed another outcome. *^a^Ae. aegypti; Ae. albopictus; Hg. janthinomys; Hg. leucocelaenus; Sa. chloropterus; Ae. luteocephalus; Ae. africanus; Ae. vittatus; Ae. niveus*. CMIP, Coupled Model Intercomparison Project; IPCC, Intergovernmental Panel on Climate Change; OS, observational study; PMS, predictive modelling study; RCP, Representative Concentration Pathways; USA, United States of America.

# Table S3 Studies on the effects of climatic factors and human behaviour on vector distribution and abundance

| **Reference** | **Study** | **Vector** | **Geography** | **Covariates** | **Key finding** |
| --- | --- | --- | --- | --- | --- |
| Carbajo 2019 | PMS/OS | *Ae. aegypti* | Argentina  *Endemic* | Air temperature, precipitation, altitude, population | Mean annual air temperature and human population were significantly associated with the past occurrence of *Ae. aegypti* (i.e., 1986–2000) and its expansion (2001–2017). The modelled distribution of *Ae. aegypti* for 2025–2030 showed a vast infestation in the north of the study area, due to increased predicted temperatures. Additionally, our results suggest there is an inhabitants’ minimum threshold for localities to harbour *Ae. aegypti* depending on the mean temperature and latitude. More densely populated areas could be infested faster and earlier, because they are more prone to receive containers with eggs through commerce or movement by citizens (e.g., Argentina). Within the context of global climatic and demographic change, the expansion of the *Aedes* mosquitoes into current free areas could be expected, with the unavoidable risk of spread of the diseases they transmit. |
| Equihua 2017 | ES/PMS | *Ae. aegypti* | Veracruz | Land change uses, biodiversity loss, urbanization without sanitation | High case numbers of dengue outside of the traditional elevation distribution limits in Veracruz, specifically during hot seasons, indicates temperature is a strong driver of the range expansion process. An increase in elevation of the potential distribution of *Ae. aegypti* because of urban development dynamics (including infrastructure, water supply, proper drainage, public services), natural ecosystem degradation and climate change could cause an expansion in the geographic distribution of dengue and other diseases transmitted by mosquitos. |
| Gorris 2024 | PMS | *Ae. aegypti;*  *Ae. albopictus;*  *Cx. erraticus;*  *Cx. nigripalpus;*  *Cx. Cx. pipiens;*  *Cx. quinquefasciatus;*  *Cx. restuans;*  *Cx. salinarius;*  *Cx. tarsalis* | North and South America  *Endemic* | Temperature and precipitation | For 2020, *Ae. aegypti* had the largest extent of high habitat suitability (>0.5) among all nine species. Mean annual temperature was in the top five most important variables for eight of the nine mosquito species – for *Cx. Salinarius*, temperature was nearly unimportant. The next most universally important variables were mean annual precipitation, boreal needleleaf evergreen trees, and temperate broadleaf deciduous shrubs. No projections for either climate change scenario indicated an eradication of any of the nine *Aedes* or *Culex* species; however, land use variables were driving projected changes in some of the mosquito distributions and is therefore an important consideration for future analyses. |
| Kearney 2009* | PMS | *Ae. aegypti;  Ae. albopictus* | Australia  *Non-endemic* | Air temperature, humidity, wind speed, rainfall and cloud cover | Climate change will directly increase habitat suitability throughout much of Australia, the potential indirect impact of changed water storage practices by humans in response to drought may have a greater effect. In northern Australia, evolutionary changes in egg desiccation resistance could potentially increase the chances of establishment in a major centre (Darwin) under climate change. |
| Khan 2020 | PMS | *Ae. aegypti;*  *Ae. albopictus* | USA and Canada  *Non-endemic* | Temperature | The northward range expansion of suitable niches for both species was projected under future climate models. Much of the USA and parts of southern Canada are projected to be suitable for both species by 2100. Under the RCP4.5 scenario, the percentage of humans living within the projected *Ae. aegypti* niche in the continental USA and Canada was projected to increase from 66% (∼186 million) in 2020 to 91% (∼364 million) by 2100. |
| Kraemer 2019 | PMS | *Ae. aegypti;  Ae. albopictus* | USA and Europe  *Non-endemic* | Human movement patterns | Significantly greater expansion is predicted, especially between 2050 and 2080, if emissions are not reduced. At the same time, future human population growth is expected to be concentrated disproportionately within areas where *Ae. aegypti* will already be established, leading to large increases in the global population at risk of diseases transmitted by these species. Overall, our predicted expansions will see *Ae. aegypti* invading an estimated 19.96 million km^2^ by 2050 (19.91–23.45 million km^2^, depending on the climate and urbanization scenarios) placing an estimated 49.13% (48.23–58.10%) of the world’s population at risk of arbovirus transmission. |
| Santos 2022* | PMS/OS | *Ae. aegypti* | Madeira  *Non-endemic* | Temperature, precipitation | Suitable areas under current climates are predicted to occur mainly in the warmer and densely inhabited coastal areas of the southern part of the island, where the species is already established. By mid-century (2041–2060), the extent of climatically suitable areas is expected to increase, mainly towards higher altitudes and in the eastern part of the island. |
| Wint 2022 | PMS | *Ae. aegypti* | Southern Europe, Mediterranean and North Africa  *Endemic* | Areas occupied by public equipment, residential housing density, human population density | The historical model matches the historical distributions well. When it is run with current climate values, the predicted present-day distribution is somewhat broader than it used to be particularly in north-west France, North Africa and Turkey. Though it is beginning to reappear in the eastern Caucasus, this ‘potential’ distribution clearly does not match the actual distribution of the species, which suggests some other factors are responsible for its absence. Future distributions based on the historical model also do not match future distributions derived from models based only on present day vector distributions, which predict little or no presence in the Mediterranean Region. At the same time, the vector is widespread in the USA which is predicted to consolidate its range there in future. |

Studies are color-coded according to whether they report (1) no expansion or regression (green), (2) low likelihood of expansion (beige), (3) high likelihood of expansion (orange). *Also assessed another outcome. ES, entomological survey; OS, observational study; PMS, predictive modelling study; USA, United States of America.

# Table S4 Studies on the effects of climatic factors on other vector parameters

| **Reference** | **Study** | **Vector** | **Geography** | **Covariates** | **Key finding** |
| --- | --- | --- | --- | --- | --- |
| Bonnin 2022 | PMS | *Ae. aegypti;  Ae. albopictus* | South-East Asia  *Non-endemic* | Air temperature, precipitation and greenhouse gas emissions | **Outcome being assessed:** *Seasonal variations on vector density and potential evolution*  For future evolutions, it is predicted that *Ae. aegypti* densities would mostly increase in comparison with contemporary levels, except for a slight decrease in central Myanmar, northern Thailand, and Cambodia at the end of the century. |
| Donnelly 2016 | PMS | *Ae. aegypti;  Ae. albopictus* | USA  *Non-endemic* | Temperature | **Outcome being assessed:** *Vector population growth rates and habitat suitability*  Much of the southern USA is suitable for *Ae. aegypti* year-round; however, a surprisingly large proportion of the USA in addition to the southern states is suitable for positive population growth for much of the year. While the amount of suitable habitat in some regions of the USA has contracted within the past 50 years, the range of suitable habitat for *Ae. aegypti* has expanded in many regions across the country. Additionally, IPCC CMIP5 model projections of future climate change suggest that climate change will reshape the range of *Ae. aegypti* in the USA, and potentially the risk of the viruses they transmit. Understanding the range of these mosquitoes should be considered a high priority for public health officials and vector control agencies. |
| Iwamura 2020 | PMS | *Ae. aegypti* | Global | Temperature and precipitation | **Outcome being assessed:** *Vector development and life cycle*  The authors’ model suggests consistent increases in suitability for the LCC of *Ae. aegypti* since 1950, both within the current range (primarily tropics, sub-tropics) and in currently marginal or uninvaded areas (primarily uninvaded subtropical, currently unsuitable temperate regions), and accelerating increases in suitability to 2050. Increases of LCC in the order of 7% (95% CI 3.1–12.4) globally were predicted by 2000 relative to a 1950s baseline, increasing to 17–24% by 2050 depending on the emissions scenario used. Furthermore, these results predict that invasion frontiers, representing expanding regions that are environmentally suitable for this species, in China and USA are predicted to advance 2.4–3.5 times faster by 2050 than was estimated through historical projections (1950–2000). Europe is expected to experience isolated areas of sustained suitability for *Ae. aegypti* in Spain, Portugal, Greece and Turkey by 2030. Globally, average temperature increases of over 1°C have already occurred since the industrial revolution, with even greater warming observed over continental land masses. The authors’ results highlight how such changes have likely already enhanced the potential for *Ae. aegypti*, if introduced, to complete its life cycle in areas with sufficient precipitation and availability of human hosts. Without drastic reduction of greenhouse gas emissions, global temperatures will continue to rise considerably over the coming decades, continuing to favour mosquito development in many regions. |
| Marinho 2016 | OS | *Ae. aegypti* | Brazil  *Endemic* | Temperature | **Outcome being assessed:** *Vector life cycles*  Projected increases will result in temperatures favourable to the *Ae. aegypti* life cycle. Local populations of *Ae. aegypti* are able to complete life cycles in extreme temperatures. |
| do Nascimento Neto 2020 | OS | *Ae. aegypti* | N/A | CO_2_ concentration, temperature and relative humidity | **Outcome being assessed:** *Vector behaviour and genetic adaptability**  In this study, the hsp26 gene showed a similar expression profile for both males and females from *A. aegypti* colonies living under the stress of the current and extreme climate scenario. Hsp26 gene expression increased in the extreme climate scenario, possibly related to a cellular response to preserve energy metabolism in adverse conditions, such as high temperature and CO_2_ concentration. However, the expression pattern was inversely proportional between males and females. There was a similar pattern of hsp83 gene expression between current and extreme climate scenarios, although the tendency of expression was mostly influenced by females, suggesting that for males, hsp83 is not involved in adapting to temperature. Similar observations were found for hsp70, although the difference in expression between sexes was not significant. The differential expression of the heat shock genes for male and female vectors living in different scenarios suggests alterations in their cellular metabolism such as mitochondria activity, resistance to desiccation, detoxification and osmotic stability. However, the influence of CO_2_ on the pattern of heat shock gene expression is still unknown and further studies are warranted. The warm edge limits of the species may not contract as quickly as assumed in most models, and the overall suitable range for the species may increase under global warming. |
| do Nascimento Neto 2024 | OS | *Ae. aegypti* | Central Amazon  *Endemic* | Temperature, humidity, CO_2_ concentration | **Outcome being assessed:** *Vector morphological changes*  A significant reduction (p<0.05) was observed mainly in the mean lengths under the current-extreme scenario (587.5 and 553.6 μm, respectively), as well as in the widths under the current-mild scenario (171 and 158.4 μm, respectively). The length of the cephalic capsule was also affected, showing significant differences in the means under the current-intermediate scenario (189.5 and 208.5 μm, respectively), as well as in the widths between the current-intermediate scenarios (173.7 and  194.9 μm, respectively). The results suggest significant changes in the morphometry of *Ae. aegypti* eggs and embryos as a result of the climatic influences to which the adults were subjected, which may have an impact on vector population density and, consequently, on arbovirus dynamics in urban environments. |
| Piovezan-Borges 2020 | PMS | *Ae. aegypti* | Brazil  *Endemic* | Temperature, CO_2_ concentration | **Outcome being assessed:** *Vector larval survivorship*  Shorter life cycles in the future will result in high numbers of mosquitoes, with potential increase in cases of diseases caused by this vector. Neither SCCS nor predation risk affected *Ae. aegypti* larval survivorship, but adult emergence pattern was affected by SCCS. Accordingly, our results did not indicate interactive effects of SCCS and predation risk on larval survivorship and emergence pattern of *Ae. aegypti* reared in SCCS in western Amazonia. *Ae. aegypti* is resistant to SCCS conditions tested, mainly due to high larval survivorship, even under extreme SCCS, and warmer scenarios increase adult *Ae. aegypti* emergence. Considering that *Ae. aegypti* is a health problem in western Amazonia, an implication of our findings is that the use of predation cues as biocontrol strategies will not provide a viable means of controlling the accelerated adult emergence expected under the IPCC climatic scenarios. |
| Portilla Cabrera 2020 | PMS | *Ae. aegypti* | Colombia  *Endemic* | Temperature, precipitation, land elevation, greenhouse emissions | For the current conditions, Colombia has ~140,612.8 km^2^ of areas with the possible presence of the vector; however, for the future, this will be reduced by more than 30%. For the future conditions, the suitable areas for A. aegypti decreased compared to the present, mainly for the year 2070 under RCP scenarios 4.5 and 8.5, however, the likelihood of mosquito occurrence increases in some departments of Colombia. Areas susceptible to the presence of A. aegypti are affected by climate change. The Caribbean and Andean regions have a high probability of mosquito distribution; therefore, control and epidemiological surveillance are required in these areas. |
| Trájer 2021 | PMS | *Ae. aegypti* | Mediterranean  *Non-endemic* | Temperature and precipitation | **Outcome being assessed:** *Vector ontogenetic development time and survival rate*  The modelled present climatic suitability patterns of the mosquito show that large areas of the lower altitude Mediterranean regions, including the coastal areas of the Balkan Peninsula, South France, and large regions of the Apennines and the Iberian Peninsulas could be suitable for *Ae. aegypti*. The future (2041–2060 and 2061–2080) projections predict the potential northward shift of the northern occurrence of the species in the circum-Mediterranean and Black Sea areas. Both, the potential development time, and survival rate of *Ae. aegypti* in the late 19th and the early 20th century could be like in the present times along the Mediterranean coast. The current climatic conditions cannot explain the absence of the mosquito in wide areas of the Mediterranean and sub-Mediterranean ecoregions. The future models predict the notable increase in the development time and survival rate of the mosquito in the southern and central regions of Europe. In general, the container ports of the Alboran, Balearic, and Aegean seas seem to be the most suitable sites for the re-colonization of the mosquito, and such northern parts of the Mediterranean Sea like the Gulf of Lion, the Ligurian, and Adriatic Seas are in less extent. |
| Williams 2014 | PMS | *Ae. aegypti* | Australia  *Non-endemic* | CO_2_ emissions, temperature | **Outcome being assessed:** *Vector bionomics*  Overall mosquito abundance is predicted to change, but results were equivocal for different climate change scenarios. *Ae. aegypti* abundance is predicted to increase under the B1 but decrease under the A2 scenario. Mosquitoes are predicted to have a smaller body mass in a future climate. Shorter extrinsic incubation periods are projected. |

Studies addressing climatic factors only and classified as listing other vector parameters (vector evolution, behaviour, or habitat) as an outcome. Studies are color-coded according to whether they report (1) no expansion or regression (green), (2) low likelihood of expansion (beige), (3) high likelihood of expansion (orange). *Also assessed another outcome. CI, confidence interval; CMIP, Coupled Model Intercomparison Project; IPCC, Intergovernmental Panel on Climate Change; LCC, life-cycle completions; OS, observational study; PMS, predictive modelling study; RCP, Representative Concentration Pathways; SCCS, Simulated Climate Change Scenarios.

# Table S5 Studies on the effects of climatic factors and human behaviour on other vector parameters

| **Reference** | **Study** | **Vector** | **Geography** | **Covariates** | **Key finding** |
| --- | --- | --- | --- | --- | --- |
| Chadee 2016 | OS | *Ae. aegypti* | Latin America and Caribbean  *Endemic* | Mosquito breeding site locations and container types, light regimen | **Outcome being assessed:** *Vector adaptive behaviours*  Within Latin America and Caribbean countries, *A. aegypti* mosquitoes are moving to underground breeding sites, such as septic tanks, demonstrating behavioural plasticity. The results of this study of vector breeding site locations, container types and effect of light regimen (continuous light, continuous dark or diurnal conditions) also suggest that *A. aegypti* breeding in water containing high organic matter within dark septic tanks are able to survive and complete their life cycle with emerging adults successfully copulating, blood feeding and ovipositing. Predicted rise in temperatures may lead to extreme weather events such as storms, droughts and fires, which may result in mosquitos seeking out oviposition containers at indoor sites. More information is needed to develop better scenarios for both the present and the future, which can be incorporated and guide transdisciplinary approaches on different aspects of and their interactions within these disease ecosystems. |
| Kearney 2009* | PMS | *Ae. aegypti;  Ae. albopictus* | Australia  *Non-endemic* | Air temperature, humidity, wind speed, rainfall and cloud cover | **Outcome being assessed:** *Vector range and habitat*  While we predict that climate change will directly increase habitat suitability throughout much of Australia, the potential indirect impact of changed water storage practices by humans in response to drought may have a greater effect. |
| Rose 2020 | PMS | *Ae. aegypti* | Africa  (African species used in study - results applicable to various global regions) | Dry season intensity and human population density | **Outcome being assessed:** *Vector evolution and behaviour*  Projected changes in relevant precipitation variables are modest and unlikely to drive substantial shifts in preference. Rapid urbanization, in contrast, is expected to result in dramatic increases in human population density and might trigger transitions to human-biting in many cities across the continent by 2050. |
| Santos 2022 | PMS/OS | *Ae. aegypti* | Madeira  *Non-endemic* | Temperature, precipitation | **Outcome being assessed:** *Vector habitat*  Minimum temperature of coldest month was the most influential climatic predictor, while human population density, residential housing density and public spaces were the most influential predictors describing land use and human settlement conditions. Suitable areas under current climates are predicted to occur mainly in the warmer and densely inhabited coastal areas of the southern part of the island, where the species is already established. By mid-century (2041–2060), the extent of climatically suitable areas is expected to increase, mainly towards higher altitudes and in the eastern part of the island. |

Studies addressing both climatic factors and human behaviour and classified as listing other vector parameters (vector evolution, behaviour, or habitat) as an outcome. Studies are color-coded according to whether they report (1) no expansion or regression (green), (2) low likelihood of expansion (beige), (3) high likelihood of expansion (orange). *Also assessed another outcome. OS, observational study; PMS, predictive modelling study.

# Table S6 Studies on the effects of climatic factors on YF infection and disease transmission

| **Reference** | **Study** | **Vector** | **Geography** | **Covariates** | **Key finding** |
| --- | --- | --- | --- | --- | --- |
| Davis 2021 | PMS | *Ae. aegypti;*  *Ae. albopictus* | Australia, China, Indonesia, the Philippines, Thailand and Vietnam  *Non-endemic* | Temperature and precipitation | For *Ae. aegypti*, decreases in SCI were evident in many regions of Thailand, Vietnam and Western Indonesia. Increases were most evident in outer border regions of Thailand and Vietnam, and in Southern and Western Indonesia. Increases were noted particularly in the Northern Philippines, and Southern and Western Indonesia. Much of the currently cold and arid regions in China’s North-Western mainland are projected to experience either no change in suitability, or a slight decrease. However, widespread increases in SCI were also observed throughout central and north-eastern regions. For *Ae. aegypti,* SCI values appear to gradually increase in most areas particularly in the southern provinces of Yunnan and Guizhou. In Australia, the difference map shows both moderate increases and moderate decreases in SCI for both vectors, across much of the country. Many arid regions of central Australia will continue to be unsuitable for both vectors between 2030 and 2050. For *Ae. aegypti*, increased suitability is projected for much of Eastern and Western Australia. Patterns fluctuate particularly for Queensland state. Slight to substantial increases were projected particularly along some, but not all, of the highly populated Eastern coaster regions of Queensland and New South Wales. Increases in suitability tend to overlap along the Eastern coastal regions, around the cities of Brisbane and Sydney, along with parts of Western Australia including the city of Perth, while the trend remains largely stable in the Southern states of Victoria and Tasmania. |
| Gaythorpe 2020 | PMS | None specified | Africa  *Endemic* | Air temperature, humidity, wind speed, rainfall and cloud cover | The Central African Republic is one of the countries most likely to see an increase in transmission, while Liberia and Guinea Bissau have more uncertain projections. This highlights that the most severe proportional increases in force of infection are seen outside West Africa. Climate change may affect not only the magnitude of YF disease burden but also its distribution. We find that, through the projected changes in both temperature and rainfall, transmission may change heterogeneously across the region. Areas with lower burden such as West Africa may have increased vaccination rates or limited increase in force of infection. Nevertheless, there is a need to implement and prepare for new vaccination activities and consolidate existing control strategies in order to mitigate the rising risk from YF. |
| Ryan 2019 | PMS | *Ae. aegypti;  Ae. albopictus* | Global | Temperature and human population density | Our results show that if mosquito range shifts track optimal temperature ranges for transmission (21.3–34.0˚C for *Ae. aegypti*), we can expect poleward shifts in *Aedes*-borne virus distributions. More severe climate change scenarios produce larger population exposures to transmission by *Ae. aegypti*. Climate-driven risk of transmission from both mosquitoes will increase substantially, even in the short term, for most of Europe. Within the next century, nearly a billion people are threatened with new exposure to virus transmission by both *Aedes spp*. in the worst-case scenario. Many other complicating factors (like mosquito range limits and viral evolution) exist; overall, our results indicate that climate change will lead to increased net and new exposures to *Aedes*-borne viruses. |
| Sadeghieh 2021 | PMS/OS | *Haemagogus spp.*  *(species)* | Brazil  *Endemic* | Temperature, precipitation | YF case projections for Brazil from the models varied when climate change scenarios were applied, including the peak clinical case incidence, cumulative clinical case incidence, time to peak incidence, and the outbreak duration. Overall, a decrease in YF cases and outbreak duration was observed. Comparing the observed incidence in 2017/18 to the projected incidence in 2070–2100, for RCP4.5, the cumulative case incidence decreased from 184 to 161, and the outbreak duration decreased from 21 to 20 weeks. For RCP8.5, the peak case incidence decreased from 184 to 147, and the outbreak duration decreased from 21 to 17 weeks. The observed decrease was primarily due to temperature increasing beyond that suitable for *Haemagogus* mosquito survival. |

Studies addressing climatic factors only and classified as listing YF infection and disease transmission as an outcome. Studies are color-coded according to whether they report (1) no expansion or regression (green), (2) low likelihood of expansion (beige), (3) high likelihood of expansion (orange). OS, observational study; PMS, predictive modelling study; SCI, Suitable Conditions Index; YF, yellow fever.

# Table S7 Studies on the effects of climatic factors and human behaviour on YF infection and disease transmission

| **Reference** | **Study** | **Vector** | **Geography** | **Covariates** | **Key finding** |
| --- | --- | --- | --- | --- | --- |
| Monaghan 2018 | PMS | *Ae. aegypti* | Global | Temperature, precipitation, greenhouse gas emission | We conclude that both climate change and population growth may increase human exposure to the virus vector mosquito *Ae. aegypti* by the late 21st century. The global population is projected to grow from year 2000 levels of 6053 million (GPW) to 11143 million for SSP3 or 8468 million for SSP5 by 2070. The percentage of this total global population exposed to *Ae. aegypti* for the reference 1950–2000 case is 63%, a value projected to increase to 68–70% by 2061–2080 if population is held constant (GPW; i.e., climate change only), to 77–80% for SSP3 and to 71–74% for SSP5. Developing economies in tropical and subtropical Africa, southern middle-income South America may be particularly impacted by the expansion of year-round *Ae. aegypti* occurrence patterns, whereas industrialized mid-latitude economies may be impacted by the expansion of seasonal occurrence patterns. The benefits of reduced anthropogenic climate change on human exposure to *Ae. aegypti* due to taking the RCP4.5 emissions scenario instead of the RCP8.5 scenario may be more pronounced in industrialized countries than in developing and middle-income countries. |
| Rohat 2020 | PMS | *Ae. aegypti;*  *Ae. albopictus* | USA  *Non-endemic* | Temperature, human behaviour,  fossil fuel emissions | In the USA, human exposure to *Aedes*-borne is projected to increase under most scenarios, up to + 177% at the national scale in 2080 under SSP5*RCP8.5 relative to a historical baseline. Projected exposure changes are predominantly driven by population changes in vulnerable demographic groups, although climate change is also important, particularly in the western region where future exposure would be about 30% lower under RCP2.6 compared to RCP8.5. The results emphasize the crucial role that socioeconomic and demographic change play in shaping future population vulnerability and exposure to *Aedes*-borne virus transmission risk in the USA and underline the importance of including socioeconomic scenarios in projections of climate-related vector-borne disease impacts. |

Studies are color-coded according to whether they report (1) no expansion or regression (green), (2) low likelihood of expansion (beige), (3) high likelihood of expansion (orange). GPW, Gridded Population of the World; PMS, predictive modelling study; RCP, Representative Concentration Pathways; SSP, Shared Socioeconomic Pathways. USA, United States of America; YF, yellow fever.

# Table S8 Studies on the effects of human behaviour on YF infection and disease transmission

| **Reference** | **Study** | **Vector** | **Geography** | **Covariates** | **Key finding** |
| --- | --- | --- | --- | --- | --- |
| Hamlet 2018 | PMS | None specified | South America  *Endemic* | Land use and habitat fragmentation | The most influential covariates on YF occurrence were habitat fragmentation, cropland, the change in cropland over time and forest cover. Cropland, along with forest cover, appeared to be protective against YF reports; however, habitat fragmentation and increases in cropland from previous years heightened the risk. This highlights a transition period where the risk of sylvatic spillover is greatest and suggests that increased habitat conversion and fragmentation will increase the risk of YF cases. |

Studies are color-coded according to whether they report (1) no expansion or regression (green), (2) low likelihood of expansion (beige), (3) high likelihood of expansion (red). PMS, predictive modelling study; YF, yellow fever
